# Building a cell-factory in *Crithidia fasciculata*: a bio-sustainable system to produce high-value polyunsaturated fatty acids

**DOI:** 10.1186/s12934-025-02760-7

**Published:** 2025-06-23

**Authors:** Michela Cerone, Louise L. Major, Terry K. Smith

**Affiliations:** Schools of Chemistry and Biology, Biomedical Science Research Complex, North Haugh, University of St Andrews, St Andrews, Fife, Scotland KY16 9ST UK

**Keywords:** Cell factory, Desaturase, Elongase, Temperature, Cooking oils, Polyunsaturated fatty acids, Bio-sustainable

## Abstract

Fatty acids (FAs) are a class of diverse biomolecules that are essential for a wide variety of biological processes. Recently, ω-3 and ω-6 polyunsaturated FAs (PUFAs) have received a lot of attention for their benefit and essentiality to human health. Their demand is constantly increasing alongside the ever-decreasing availability of sources of natural PUFAs, such as some plants and marine ecosystems, which are now endangered by agribusiness and climate change. Most of the novel microbial synthetic platforms to produce ω-3 and ω-6 PUFAs (e.g. yeast, microalgae, oleaginous microorganisms, bacteria etc.) are costly. On the other hand, industrial processes based on chemical total synthesis of PUFAs is often challenging. Here, we propose an innovative and bio-sustainable method to make ω-3 and ω-6 PUFAs by building a cell-factory in *Crithidia fasciculata*, using genetic manipulation of endogenous FA desaturases and elongases, supplementation of the culture media with cheap sources of fats (such as used cooking oils), and culturing temperature reduction. In these unusual environments, *C. fasciculata* show high adaptability: the cells grow rapidly producing large biomass from small volumes of culture, scavenge and remodel unusual fat sources, as well as biosynthesising high value FAs and PUFAs *de novo*. By genetic manipulation, allowing overexpression of either the endogenous Δ6-desaturases or Δ4-desaturases, in conjunction with the endogenous elongase Elo4, high value ω-6 and ω-3 PUFAs were yielded to a maximum of 50% of the total FA content of the cells. These bioengineered *C. fasciculata* provide a low-cost and bio-sustainable platform to increase the production of essential PUFAs which can be introduced into the food chain at lower costs.

**Supplementary Information:**

The online version contains supplementary material available at 10.1186/s12934-025-02760-7.

## Introduction

Fatty acids (FAs) are essential biomolecules in all living organisms. FAs are carboxylic acids with a long and hydrogenated carbon chain, containing from a minimum of 8 to a maximum of 30 carbons [1–3]. According to the level of unsaturation of the carbon chain, FAs can be classified as either saturated (SAFAs), with no double bonds (i.e. myristic acid, 14:0), unsaturated (UFAs), with at least one double bond (i.e. oleic acid, 18:1), or polyunsaturated (PUFAs) (i.e. arachidonic acid, 20:4), with multiple double bonds (up to 6 double bonds) [1–3]. Naturally occurring and biologically active UFAs and PUFAs are those containing double bonds in the *cis-*configuration [1, 2, 4]. These type of UFAs and PUFAs are the major component of biological membranes, sources of a variety of lipid signalling molecules, and therefore they participate to many biological processes [2, 5, 6]. Due to their key roles in many physiological and pathological pathways, PUFAs are considered essential molecules for human and animal health, especially those classified as ω-3 and ω-6 PUFAs [7, 8]. However, humans and animals cannot produce these essential PUFAs (EPUFAs) *de novo*, thus having to scavenge them from dietary intake, fortified foods and supplements [9, 10]. As a consequence, there is an ever-increasing terrestrial demand of EPUFAs worldwide. At the same time, the most abundant reservoirs of EPUFAs, such as the marine ecosystem and certain plants, are rapidly diminishing due to climate change and agribusiness [11, 12]. Therefore, alternative routes for the production of PUFAs are being explored [13]. Currently, the two main approaches are based on either total chemical synthesis or microbial biosynthesis using cell factories [14–16]. In the first case, PUFAs are often obtained using very complex multi-step synthetic routes, such as cross-coupling or Wittig reaction, which are laborious and challenging. In fact, they have a limited substrate scope, as they are often based upon fossil fuel derivatives, they show low *cis*-stereoselectivity, as well as poor catalyst activity and phenomena of over-reduction. Furthermore, they can have a significant environmental impact caused by the use of organic solvents and toxic reagents [17–20]. The microbial biosynthesis, instead, has many advantages. Firstly, PUFAs are synthesised through biocatalytic pathways by highly specialised enzymes, namely desaturases, which insert double bonds along the FA carbon chain, and elongases, which add 2-carbon unit on the non-reducing end of the FA at each cycle [4]. These enzymes can use a wide variety of naturally occurring substrates to exclusively obtain *cis*-PUFAs, in an aqueous environment and under mild conditions. Canonical microbial cell factories for the synthesis of PUFAs are bioengineered plants (i.e. *Camelina sativa*), microalgae (i.e. *Isochrysis galbana*), and oleaginous microorganisms (i.e. *Yarrowia lipolytica*) [21–23]. These systems can yield large amounts of PUFAs at both small and large scale; however, they require temperature, pH and light controlled environments, and/or expensive media supplements for their growth [24]. Consequently, there is an urgent need to exploit alternative platforms for a cheaper, greener and more bio-sustainable production of EPUFAs [25]. Herein, we propose the use of bioengineered *Crithidia fasciculata* to obtain high-value PUFAs. *C. fasciculata* is a single cell, eukaryotic, non-vertebrate kinetoplastid parasite, which can grow on cheap media at ambient temperatures (Figure S1A) [26–29]. Furthermore, *C. fasciculata* are formidable producers of a wide variety of FAs, especially of ω-6 and ω-3 PUFAs, thanks to their vast repertoire of FA desaturases and elongases [28]. In our previous study, we highlighted *C. fasciculata’s* plasticity and adaptability, which are some of the most desirable characteristics of an efficient cell-factory [28]. Particularly, we showed that *C. fasciculata* can grow in basic media with or without supplementation with cheap cooking oils. In this unusual environment, the cells scavenge and utilise the carbon sources available from the oils’ FA mixture to produce larger amounts of PUFAs [28]. Under these conditions, the cells grow rapidly at ambient temperature to a very high cell density, producing a relatively large biomass from small volumes of culture [28]. Based upon this, we decided to exploit our cell system further by combining chemical supplementation and genetic manipulation of endogenous FA desaturases and elongases to obtain even higher levels of PUFAs. Considering the well-known effect of the temperature on membrane fluidity, we also introduced the temperature variable into the system [30]. Here, we describe how, by regulating the temperature, supplementing the media with cheap cooking oils, and overexpressing either the endogenous Δ6- (CFAC1_280079900.1) or Δ4- (CFAC1_110026100.1) desaturases, in conjunction with the endogenous elongase Elo4 (CFAC1_110016500.1) [31], significant amounts of useful ω-3 PUFAs can be produced at low-cost via a bio-sustainable approach.

## Results

### Co-overexpression of either the endogenous Δ6 or Δ4 desaturases with Elo4 elongase in *C. fasciculata*

To up-regulate the synthesis of PUFAs in *C. fasciculata*, we designed our cell-factory to overexpress either the endogenous Δ6-desaturase (CFAC1_280079900.1) (Cf-D6) or Δ4-desaturase (CFAC1_110026100.1) (Cf-D4) in conjunction with the endogenous Elongase-4 (CFAC1_110016500.1) (Cf-Elo4) [31], using the pNUS-ss-MCS-C-term-V5-Hyg vector for Cf-Δ6 and Cf-D4 and pNUS-ss-MCS-C-term-V5-Neo for Cf-Elo4. Both vectors were derived from the one described by Tetaud et al., but modified to improve and accelerate the translation of our target genes. We replaced the CfPGK 5’ UTR adjacent to the Hyg cassette with the *Cf*EAP1 gene 5’UTR and introduced a new multiple cloning site, containing a secretory signal and a V5-tag at the C-terminus (Figure S1B) [29, 32]. Initially, three different overexpression cell lines were obtained OE-D6, OE-D4 and OE-ELO4, each of them expressing *Cf-D6*,* Cf-D4* and *Cf-Elo4* as episomal copies [29]. Finally, OE-D6 and OE-D4 mutants were transfected with pNUS-ss-Elo4-C-term-V5-Neo obtaining OE-D6-ELO4 and OE-D4-ELO4 cell-factories (Figure S2A-C). The plasmid integration was confirmed by PCR using episomal plasmid extracted from the mutants (Figure S3).

### The cellular location of Cf-Δ6, Cf-Δ4 and Cf-Elo4 in *C. fasciculata*

**Fig. 1:**
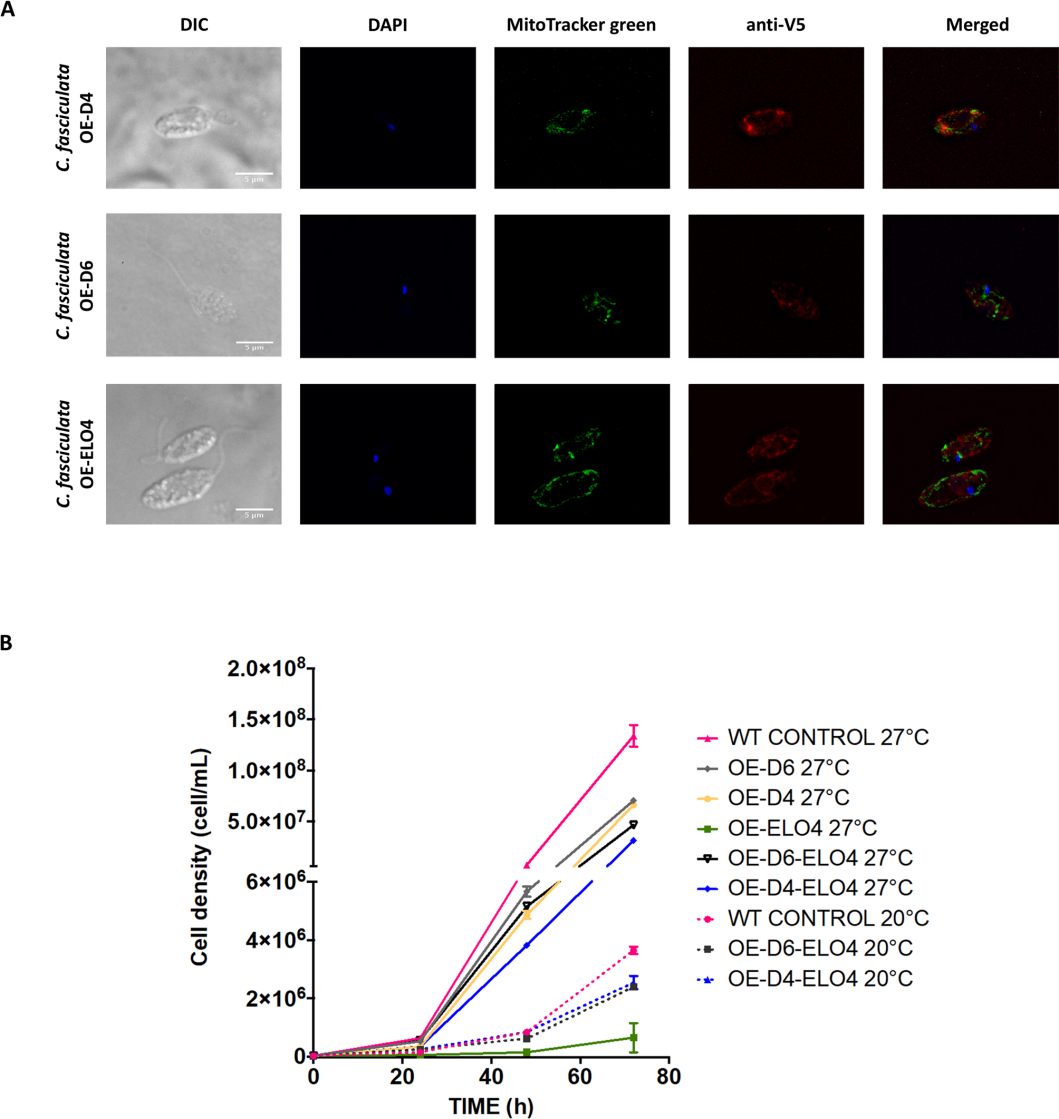
Cf-Δ6, Cf-Δ4 and Cf-Elo4 cellular localisation in *C. fasciculata*. Immunofluorescence microscopy images of *C. fasciculata* OE-D4 (first row), OE-D6 (second row) and OE-ELO4 (third row) fixed on poly-lysine coated slides stained with DAPI (blue signal), MitoTracker^®^ green (green signal), anti-V5 tag (red signal) and imaged with Deltavision confocal microscope. The cells were grown for 72 hors in standard media at 27˚C. The images show that Cf-Δ6 and Cf-Δ4 (anti-V5 tag red dots) are partially located in some parts of the mitochondrial compartments (green). Cf-Elo4 (anti-V5 tag red dots) is partially located in the mitochondrial compartment and partially in the ER. The scale bar is 5 μm. **B**) Growth curves of OE-D6, OE-D4 and OE-ELO4 *C. fasciculata* cell-factories at 27˚C and 20˚C. The graph represents the growth curves over 72 h of *C. fasciculata* WT control and *C. fasciculata* Cf-Δ6 (OE-D6), Cf-Δ4 (OE-D4) and Cf-Elo4 (OE-ELO4) overexpression, and *C. fasciculata* co-overexpressing Cf-Δ6 and Cf-Elo4 (OE-D6-ELO4) and Cf-Δ4 and Cf-Elo4 (OE-D4-ELO4), when the cells are cultured in standard media at 27˚C (solid lines) and 20˚C (dashed lines) (only WT, OE-D6-ELO4 and OE-D4-ELO4). Values are the mean of three independent biological replicates (*n* = 3). Error bars represent the standard deviation of each mean. Statistical analysis was performed by PRISM 6 using One-way ANOVA multiple comparisons based on a Tukey t-test with a 95% confidence interval

The cellular location of Cf-Δ6, Cf-Δ4 and Cf-Elo4 was also determined taking advantage of the fact that the elongase and desaturases are expressed as C-terminal-V5-tagged proteins. OE-D6, OE-D4, OE-ELO4 and WT cells were cultured for 48 h under appropriate drug selection and then harvested at mid-log phase of their growth. Initial analysis by immunoblotting showed that Cf-D6, Cf-D4, Cf-ELO4 are over expressed in *C. fasciculata* (Figure S4). However, they did not migrate exactly according to their predicted size (~ 48, 49 and 36 kDa, respectively), suggesting potential dimers formation. The Cf-D6-V5, Cf-D4-V5, Cf-ELO4-V5 proteins were also detected by immunofluorescence microscopy using an anti-V5 secondary antibody in the presence of MitoTracker™ Green and DAPI stains. The red, fluorescent punctate signals from Cf-D6-V5 and Cf-D4-V5 were both partially localised/associated with parts of the mitochondria (green fluorescence) in the cells (Fig. 1A). The red, fluorescent and punctate signals from Cf-ELO4-V5 seemed to be distributed in the endoplasmic reticulum (ER), as it surrounds the nucleus, but also in some parts of the mitochondria, suggesting a possible association with the ER-mitochondria contact sites. We believe that this is important to know the locations of these and other associated enzymes that will have to work in tandem to produce the desired fatty acids, and therefore having the same organellar / cellular location is highly desirable.

### The growth rate of the cell factories is temperature dependent

To ensure that the cell-factories could still divide rapidly after genetic manipulation, growth curves were measured over 72 h at standard culturing temperature of 27˚C, but also at 20˚C. After 72 h at 27˚C, the WT control reached a very high cell-density (Fig. 1B), which was significantly higher than that of all the cell factories (*p* < 0.0001). Despite that, the growth trend of all the cell factories, except for OE-ELO4, was maintained identical to the WT throughout the first 48 h, with a higher final cell density ranging between 3 and 5 × 10^7^ cells/mL. This suggests that the cells can adapt to the genetic manipulations and form a high level of biomass, after a process of metabolic adaptation, which is most evident between 24 and 48 h time points, when their growth is slower. Interestingly, when the Cf-Elo4 was overexpressed without the desaturases, the cells showed a very significant lag in the growth rate between 24 and 48 h time points. After this static phase, OE-ELO4 cells recovered their growth rate. However, this was 3 orders of magnitude lower than the WT and all the other cell factories. The data clearly shows that when Cf-Elo4 is overexpressed, Cf-Δ6 and Cf-Δ4 must also be upregulated for the cells to adapt and grow at a rate similar to the WT. This is also in line with the biochemical function of desaturases and elongases, which must work in tandem to synthesise the correct PUFAs and ensure the correct membrane fluidity, hence cell viability [4]. Upon this observation, we decided to record the growth of only OE-D6-ELO4 and OE-D4-ELO4 cells at 20˚C. After 72 h, the cell-factories and the WT control adapted to the lower temperature, despite a reduction in their final cell density by almost a factor of 100-fold compared to the cells grown at 27˚C. This response is expected when considering that the cells are growing at a lower temperature and under increased membrane rigidity, which they need to rebalance. Hence, they slow their growth while tuning the FAs metabolism to maintain the correct ratio of SFAs and UFAs. Nevertheless, OE-D6-ELO4 and OE-D4-ELO4 reached a reasonably high final density of ~ 2.5 × 10^6^ cell/mL. It is also evident that the growth trend has not reached the steady state yet, suggesting that even under this condition the OE-D6-ELO4 and OE-D4-ELO4 may reach even higher density, which would be visible by monitoring the growth for longer than 3 days.

### The effect of the temperature and of the genetic manipulations on the fatty acid profile of *C. fasciculata*

By overexpressing the endogenous desaturases Cf-Δ6 and Cf-Δ4 and the elongase Cf-Elo-4 in *C. fasciculata*, we were expecting to see an overall increase in the levels of UFAs and long chain UFAs. To test our hypothesis, we used GC-MS to analyse the total FA content of the cell factories and the WT control at 72 h at either 27˚C or 20˚C (Figure S5, S6 and S7).

**Fig. 2:**
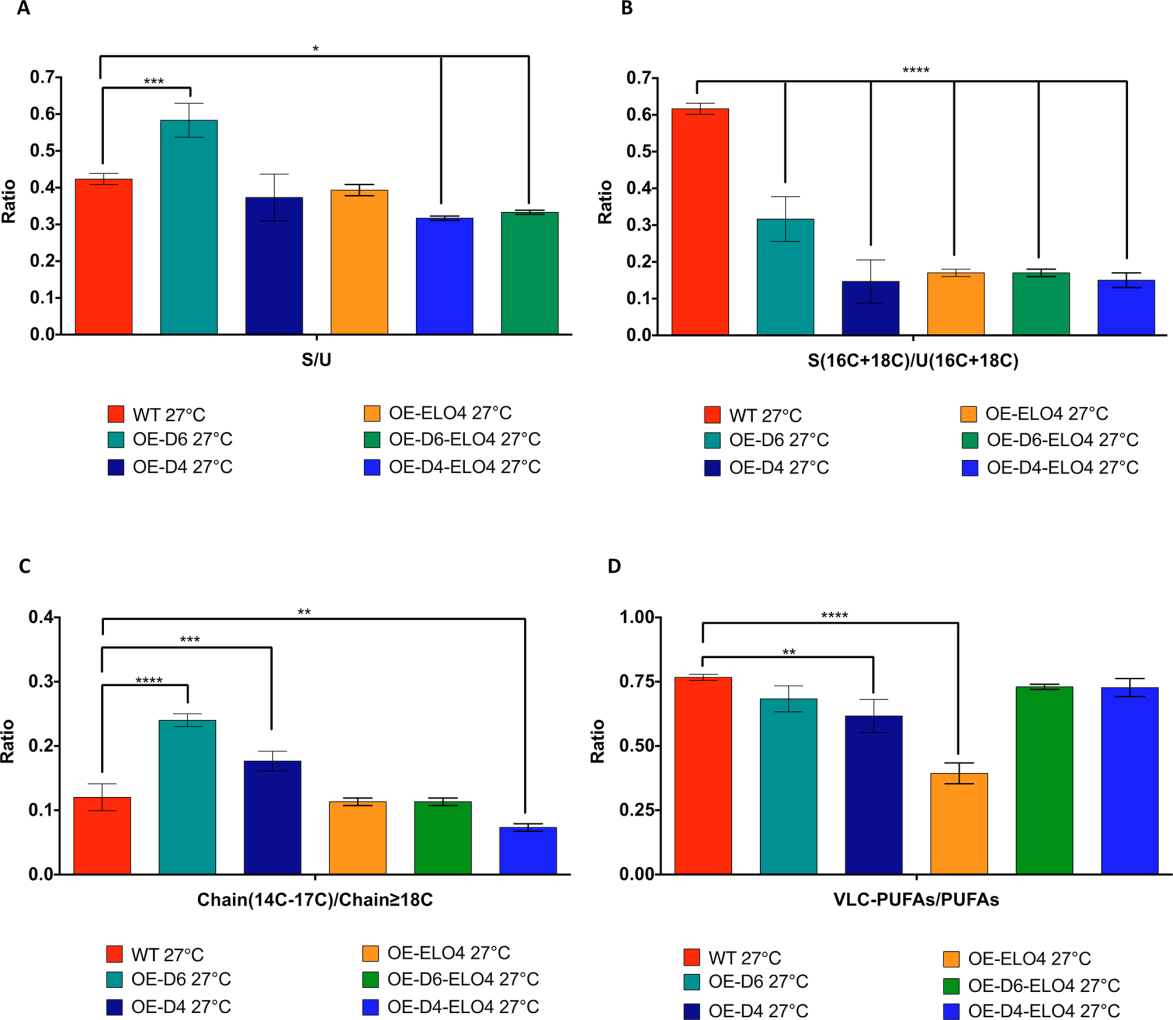
The ratio between different species of FAs produced by *C. fasciculata* cell-factories at 27˚C considering the level of unsaturation and chain length. The bar charts show (**A**) the ratio between the total amount of SAFAs (S) versus the total amount of the UFAs (U), (**B**) the ratio between the total amount of SAFAs (S(16 C + 18 C)) versus the total amount of the UFAs (U(16 C + 18 C)), (**C**) the ratio between the total amount of FAs with less then 18 C on the chain (Chain(14–17 C)) versus the total amount of the FAs with 18 C or more on the chain (Chain(≥ 18 C)), (**D**) the ratio between the total amount of very long chain PUFAs (VLC-PUFAs) versus the total amount of the shorter chain PUFAs (PUFAs). These values are obtained from OE-D6, OE-D4, OE-ELO4, OE-D6-ELO4 and OE-D4-ELO4 *C. fasciculata* cell-factories and WT control cultured in standard media at 27˚C, as indicated in the legend. Values are the mean of three independent biological replicates (*n* = 3). Error bars represent the standard deviation of each mean (±). All FAs were identified using GC-MS based upon retention time, fragmentation, and comparison with standards. Statistical analysis was performed by PRISM 6 using One-way ANOVA multiple comparisons based on a Tukey t-test with a 95% confidence interval

**Fig. 3:**
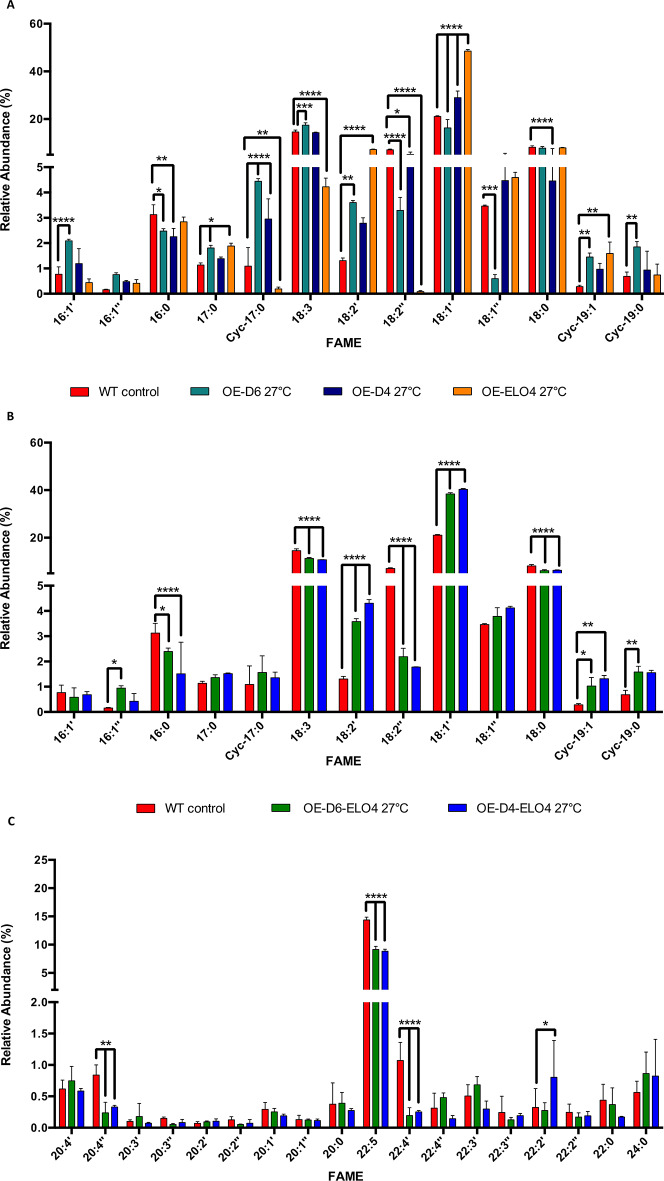
GC-MS analysis of FAs produced in OE-D6, OE-D4, OE-ELO4, OE-D6-ELO4 and OE-D4-ELO4 *C. fasciculata* cell-factories grown in standard media at 27˚C. **A**) The bar chart shows the difference in 16 C, 17 C, 18 C and 19 C FAs (X axis, the order follows increasing retention time) and the relative abundance (Y axis) found in *C. fasciculata* overexpressing Cf-Δ6 (OE-D6), Cf-Δ4 (OE-D4) or Cf-Elo4 (OE-ELO4) and WT control, grown in standard media at 27˚C, as shown in the legend. **B**) The bar chart shows the difference in 16 C, 17 C, 18 C and 19 C FAs (X axis, the order follows increasing retention time) and the relative abundance (Y axis) found in *C. fasciculata* overexpressing Cf-Δ6 or Cf-Δ4 in conjunction with Cf-Elo4 (OE-D6-ELO4 and OE-D4-ELO4) and WT control, grown in standard media at 27˚C, as shown in the legend. **C**) The bar chart shows the difference in VLC-PUFAs (X axis, the order follows increasing retention time) and the relative abundance (Y axis) found in *C. fasciculata* overexpressing Cf-Δ6 or Cf-Δ4 in conjunction with Cf-Elo4 (OE-D6-ELO4 and OE-D4-ELO4) and WT control, grown in standard media at 27˚C, as shown in the legend. Values are the mean of three independent biological replicates (*n* = 3). Error bars represent the standard deviation of each mean (±). All FAs were identified using GC-MS based upon retention time, fragmentation, and comparison with standards. Statistical analysis was performed by PRISM 6 by using One-way ANOVA multiple comparisons based on a Tukey t-test with a 95% confidence interval

At 27˚C we observed a lower ratio between SAFAs and UFAs (S/U) in both OE-D6-ELO4 (p = 0.0318) and OE-D4-ELO4 (p = 0.0104) cell factories compared to the WT control (Fig. 2A). This implies that the UFA content is significantly higher in the cell-factories (Fig. 2A). Unexpectedly, when only Cf-Δ6 was overexpressed, we detected an increase in S/U ratio, caused by a higher level of SAFAs, and particularly of cyc-17:0 and cyc-19:0 (Fig. 3A). These cyclic SAFAs are normally produced when higher amounts of PUFAs, in this specific case 18:3 and 18:2 (Fig. 3A), are present in the membranes, in order to maintain the correct fluidity [33]. No significant changes in S/U were observed at 27˚C, when only Cf-Δ4 or the Cf-Elo-4 were overexpressed. However, the levels of 16 C and 18 C UFAs, were significantly higher in all cell factories compared to the WT (p < 0.0001) (Fig. 3A). Unexpectedly, no difference in the level of long chain FAs (≥ 18 C) was detected in OE-ELO4 cells compared to the WT control (Fig. 2C). Whereas an increase in long chain FAs was observed when the cells were overexpressing the Cf-Δ4 in conjunction with Cf-Elo-4 (p = 0.0023). A decrease was instead detected in OE-D6 and OE-D4 cell-factories compared to the WT control (p < 0.0001). These results suggest that at 27˚C, if the level of 16 C and 18 C UFAs increases due to the overexpression of Cf-Δ4 or Cf-Δ6, so does the level of shorter chain SAFAs (Fig. 2B and C). This is essential for the cells to maintain the appropriate membrane fluidity [30]. Furthermore, to see an increase in both long chain FAs and UFAs, such as 18:1’ and 18:2’, Cf-Δ4 and Cf-Elo-4 must be overexpressed at the same time (Figs. 2B and C and 3C). Finally, looking at the ratio of very long chain PUFAs (VLC-PUFAs) (20–22 C) and PUFAs (18 C), no differences were detected between the WT and the OE-D6-ELO4 or OE-D4-ELO4 cell factories at 27˚C (Figs. 2D and 3C).

**Fig. 4:**
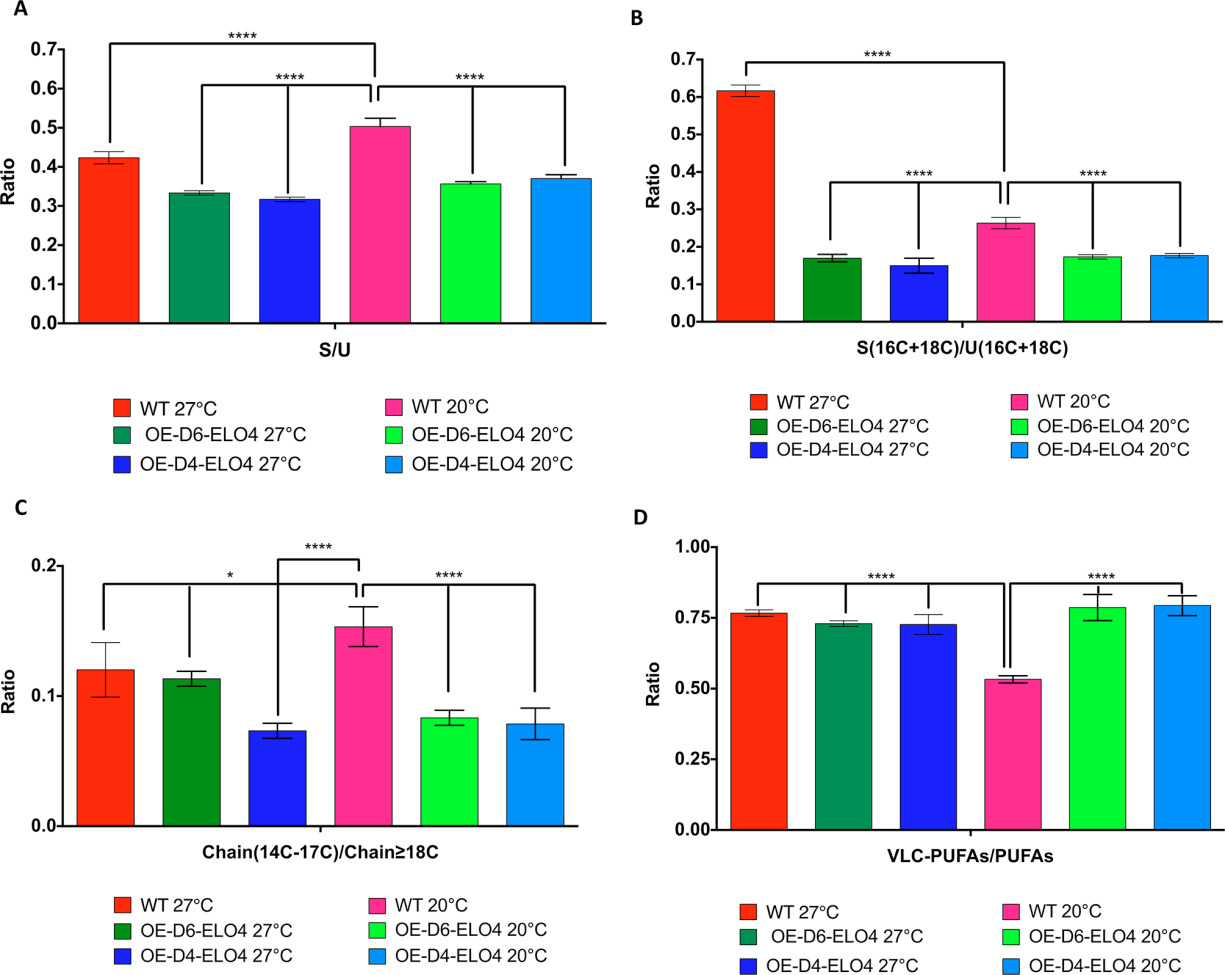
The ratio between different species of FAs produced by *C. fasciculata* cell-factories at 20˚C considering the level of unsaturation and chain length. The bar charts show (**A**) the ratio between the total amount of SAFAs (S) versus the total amount of the UFAs (U), (**B**) the ratio between the total amount of SAFAs (S(16 C + 18 C)) versus the total amount of the UFAs (U(16 C + 18 C)), (**C**) the ratio between the total amount of FAs with less then 18 C on the chain (Chain(14–17 C)) versus the total amount of the FAs 18 C or more on the chain (Chain(≥ 18 C)), (**D**) the ratio between the total amount of very long chain PUFAs (VLC-PUFAs) versus the total amount of the shorter chain PUFAs (PUFAs). These values are obtained from OE-D6, OE-D4 and OE-ELO4 *C. fasciculata* cell-factories and WT control cultured in standard media at 20˚C, as indicated in the legend. Values are the mean of three independent biological replicates (*n* = 3). Error bars represent the standard deviation of each mean (±). All FAs were identified using GC-MS based upon retention time, fragmentation, and comparison with standards. Statistical analysis was performed by PRISM 6 using One-way ANOVA multiple comparisons based on a Tukey t-test with a 95% confidence interval

**Fig. 5:**
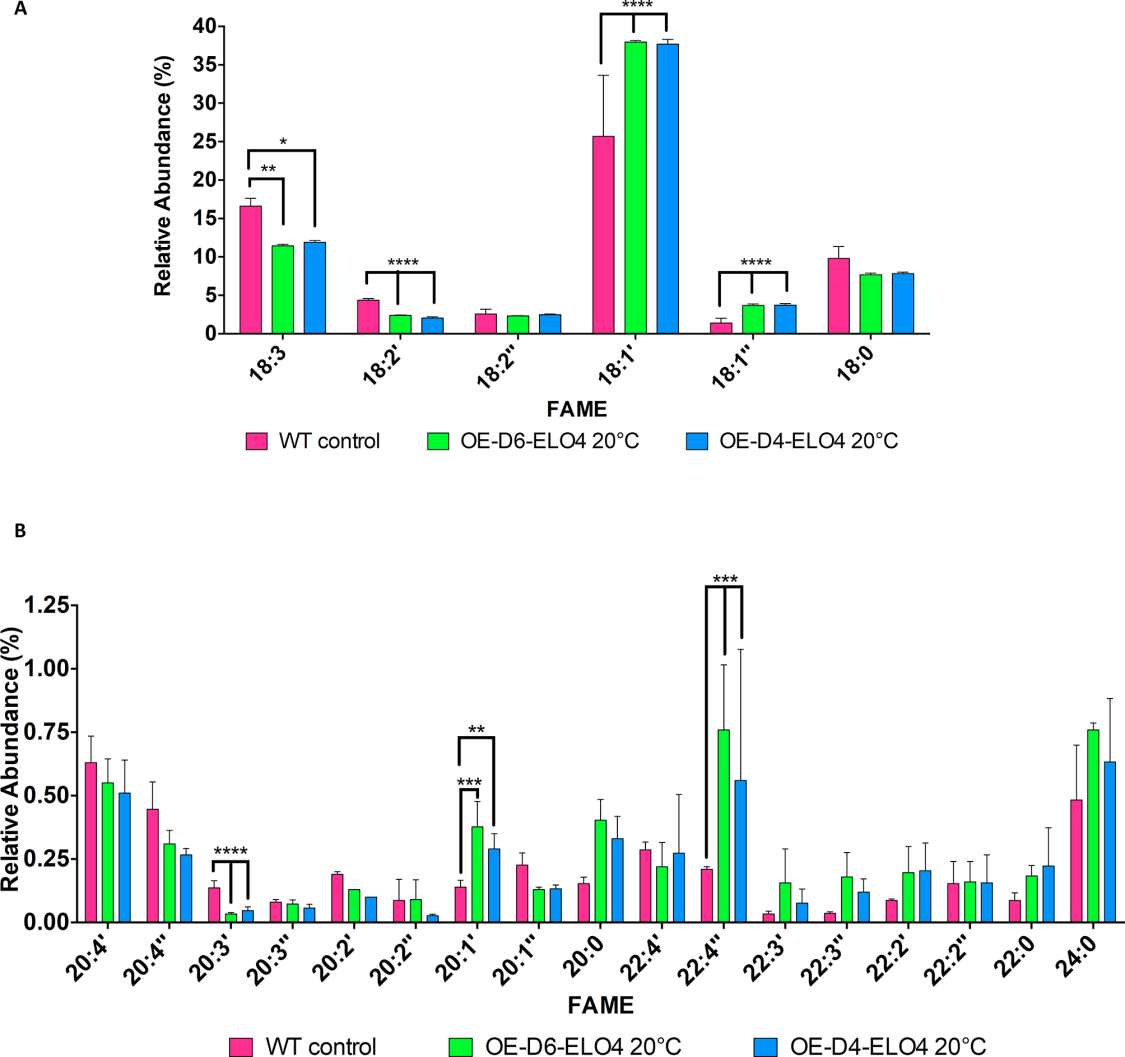
GC-MS analysis of FAs produced in OE-D6-ELO4 and OE-D4-ELO4 *C. fasciculata* cell-factories grown in standard media at 20˚C. **A**) The bar chart shows the difference in 18 C FAs (X axis, the order follows increasing retention time) and the relative abundance (Y axis) found in *C. fasciculata* overexpressing Cf-Δ6 or Cf-Δ4 in conjunction with Cf-Elo4 (OE-D6-ELO4 and OE-D4-ELO4) and WT control, grown in standard media at 20˚C, as shown in the legend. **B**) The bar chart shows the difference in VLC-PUFAs (X axis, the order follows increasing retention time) and the relative abundance (Y axis) found in *C. fasciculata* overexpressing Cf-Δ6 or Cf-Δ4 in conjunction with Cf-Elo4 (OE-D6-ELO4 and OE-D4-ELO4) and WT control, grown in standard media at 20˚C, as shown in the legend. Values are the mean of three independent biological replicates (*n* = 3). Error bars represent the standard deviation of each mean (±). All FAs were identified using GC-MS based upon retention time, fragmentation, and comparison with standards. Statistical analysis was performed by PRISM 6 by using One-way ANOVA multiple comparisons based on a Tukey t-test with a 95% confidence interval

However, an increase of 18 C PUFAs and in 22:2’ VLC-PUFA was observed when either the Cf-Δ4 (*p* = 0.0033) or the Cf-Elo-4 (*p* < 0.0001) were overexpressed (Figs. 2D and 3B) compared to the WT (Fig. 3C). Rather surprisingly, there were no significant differences in the various ratios between the OE-D6-ELO4 and OE-D4-ELO4 cell factories grown at either 20˚C or 27˚C (Fig. 4). In contrast differences were observed for the WT between 20˚C and 27˚C (Fig. 4). The S/U ratio was significantly lower in the WT at 27˚C than at 20˚C, due to an overall higher level of UFAs (*p* < 0.0001) (Fig. 4A). However, the amount of 16 C and 18 C UFAs was significantly higher at 20˚C, hence the lower ratio S (16 C + 18 C)/U (16 C + 18 C) observed (*p* < 0.0001) (Figs. 4B and 5A). This suggests that the cells selectively produce more UFAs of medium-chain length to improve membrane fluidity at this lower temperature. Long chain FAs (18 C or more) were more represented at 27˚C (hence lower ratio Chain (14–17 C)/Chain ≥ 18)) (*p* < 0.0001) (Fig. 4C), of which almost 15% was made of ω3–22:5 PUFAs (Figure S5, S6 and S7). This was confirmed when looking at the ratio between VLC-PUFAs/PUFAs, which was in fact higher in the WT at 27˚C (*p* < 0.0001) (Fig. 4D). This ratio was the same for the cell factories both at 27˚C and 20˚C, whereas it was significantly lower for the WT at 20˚C (*p* < 0.0001), confirming that the cells prefer to produce more 18 C UFAs under these conditions (Fig. 4D). It is important to highlight that OE-D6-ELO4 or OE-D4-ELO4 cell factories produced the same level of VLC-PUFAs at 20˚C, and particularly they made larger amounts of 22:4’’ and 20:1’ VLC-PUFAs (Fig. 5B). Overall, by tuning the temperature and the level of expression of endogenous Cf-Δ6, Cf-Δ4 and Cf-Elo-4, the most abundant PUFAs and VLC-PUFAs detected in all the cell lines were ω3–18:3 (α-linolenic acid), and its precursor 18:1 (oleic acid), and the ω3–22:5 (docosapentaenoic acid) and the ω6–22:4 (adrenic acid) (Fig. 5A-B; Figure S5, S6 and S7).

### The use of cooking oils as cheap sources of FA Building blocks to produce high-value PUFAs in *C. fasciculata*

**Fig. 6:**
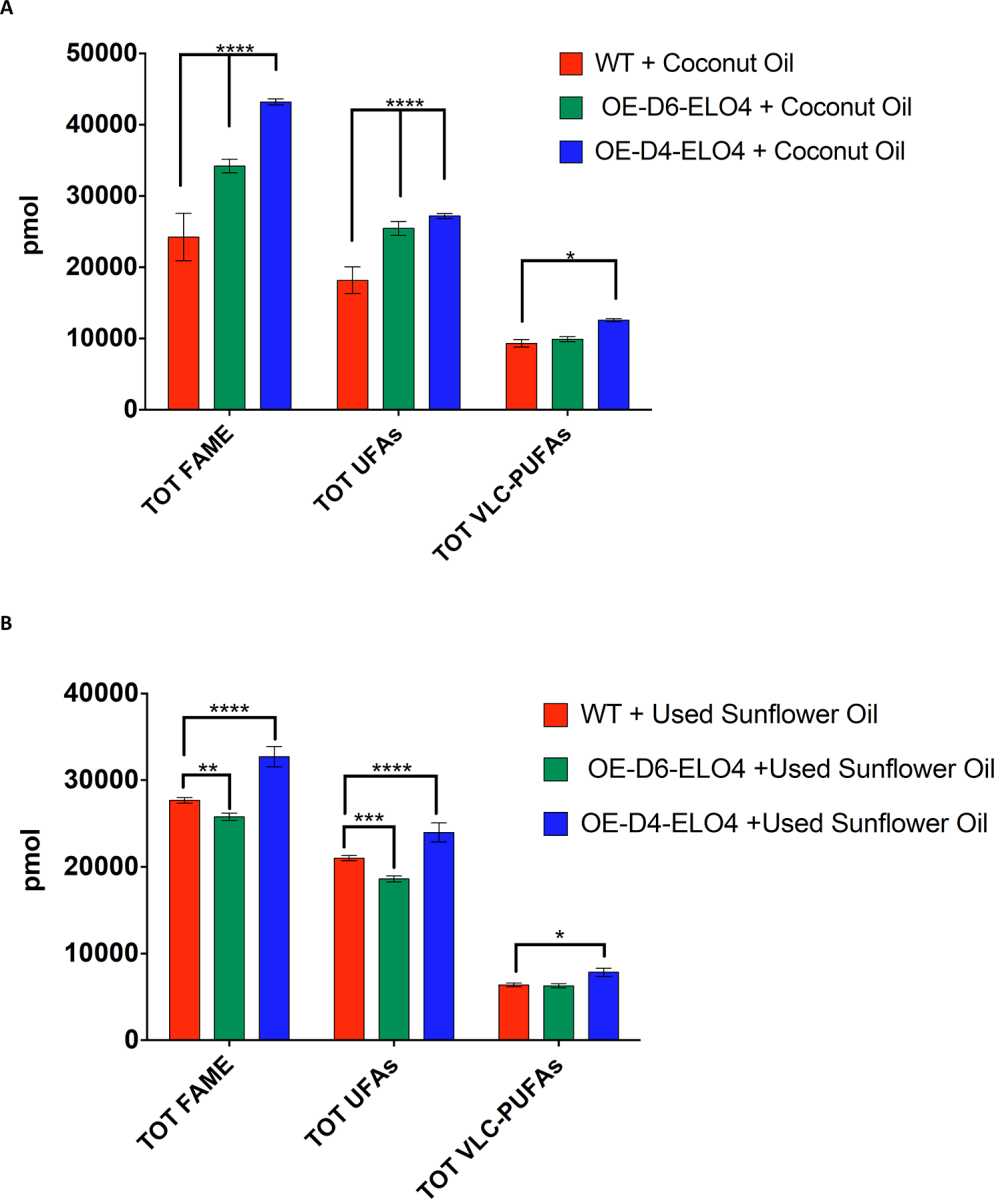
The total amount of FAs, UFAs and VLC-PUFAs produced by the cell factories and WT upon coconut oil and used sunflower oil supplementation at 27˚C. The bar charts show the total amount FAs (TOT FAME), the total amount of the UFAs (TOT UFAs) and the total amount of VLC-PUFAs (TOT VLC-PUFAs) produced by OE-D6-ELO4 and OE-D4-ELO4 *C. fasciculata* cell-factories and WT control cultured in media supplemented with coconut oil (**A**) and used sunflower oil (**B**) at 27˚C, as indicated in the legend. Values are normalised against the D27-myristate internal standard present in each sample at 48 pmol per 1U of area under the peak. Values are the mean of three independent biological replicates (*n* = 3). Error bars represent the standard deviation of each mean (±). All FAs were identified using GC-MS based upon retention time, fragmentation, and comparison with standards. Statistical analysis was performed by PRISM 6 using One-way ANOVA multiple comparisons based on a Tukey t-test with a 95% confidence interval

So far, we have shown that *C. fasciculata* can produce large amounts of FAs, and in particular ω3- and ω6-PUFAs. The cells also exhibited the ability to tune the biosynthesis of these PUFAs, as well as UFAs and SAFAs, to respond to varying levels of expression of their endogenous desaturases and elongases, as well as to the change in temperature. Based upon data from our previous study, we know that WT *C. fasciculata* can efficiently uptake and remodel FA sources from cheap and commercial cooking oils, to make a large amount of 18 C UFAs [28]. Therefore, we decided to try and push the cell factories further to try and produce even larger amounts of high-value PUFAs, by supplementing the media with two of the oils tested in our previous work, namely coconut oil and used sunflower oil [28]. The two oils have different levels of SAFAs and UFAs, which can be tested as substrates for the desaturases and the elongase [28]. Particularly, coconut oil was chosen based upon data from our previous work, showing that 12:0 and 14:0 SAFAs highly represented in this oil are efficiently remodelled by WT cells to produce high levels of 18 C UFAs [28]. On the other hand, used sunflower oil was selected not only because rich in 18 C UFAs, but also with the idea of recycling a very common oil waste [16, 28]. Thus, OE-D6-ELO4, OE-D4-ELO4 and the WT control *C. fasciculata* in the oil-containing media were cultured at 27˚C for 4 days to possibly obtain a larger biomass. After this period, the cells were harvested, and 1 g of wet-cell pellet was collected from each culture to which 250 pmol of internal standard D_27_-myristic acid was added to allow quantitative GC-MS analysis of the total FA pool. We determined the absolute amount of the total FAs, UFAs and VLC-PUFAs produced by the WT cells and by the cell-factories (OE-D6-ELO4 and OE-D4-ELO4) supplemented with coconut oil (Fig. 6A). Both cell-factories yielded significantly more total FAs, as well as UFAs, compared to the WT control in the same condition (*p* < 0.0001). The yield of the VLC-PUFAs was also significantly increased in OE-D4-ELO4 cells compared to the WT (*p* = 0.0203) (Fig. 6A). The supplementation with sunflower oil showed better results for the OE-D4-ELO4 cells (Fig. 6B). In fact, the yield of the total FAs produced by this cell-factory was 1.2-fold (*p* < 0.0001) greater than the WT (Fig. 6B), whereas OE-D6-ELO4 cells produced around 1.1-fold (*p* = 0.0028) less FAs than the WT control (Fig. 6B). Equally, OE-D4-ELO4 cells displayed a better utilisation of the used sunflower oil, showing higher yield of UFAs (*p* < 0.0001) and of VLC-PUFAs (*p* = 0.0189) compared to the WT control. Upon these supplementations and temperature, OE-D6-ELO4 cells showed instead a reduction in the total UFAs (*p* = 0.0003), and no changes in the total of VLC-PUFAs compared to the WT control (Fig. 6B). Clearly, the FA components in the oils and the selectivity of Cf-Δ6, Cf-Δ4 and Cf-Elo4 towards those building blocks (or substrates), are determining factors to increase the yield of UFAs and VLC-PUFAs produced by the cell-factory at 27˚C.

## Discussion

**Fig. 7:**
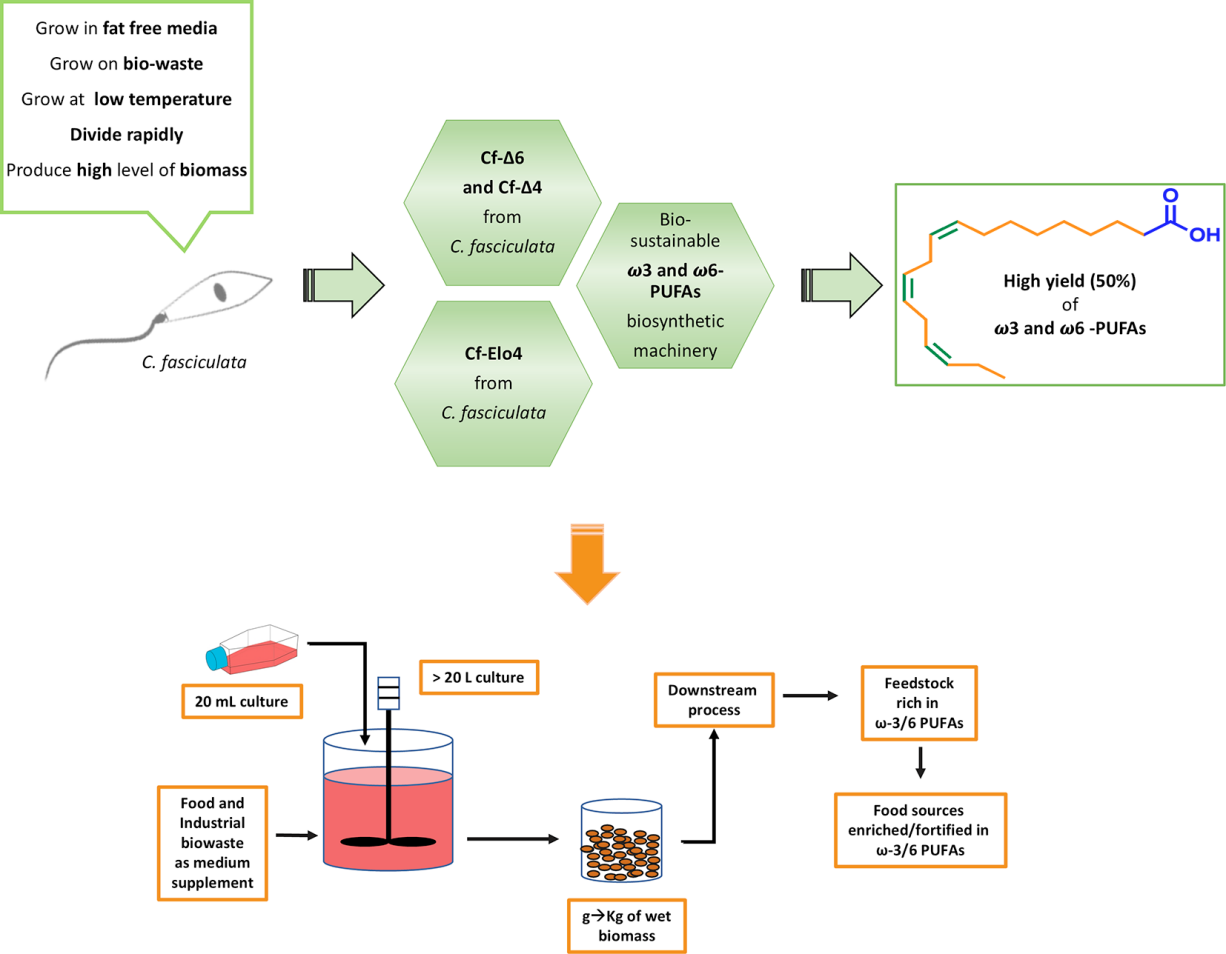
Design, results and future applications of the PUFA cell-factories in *C. fasciculata*. A schematic representation of approach to design a PUFA cell-factory in *C. fasciculata* (light green, top), and of the PUFA cell-factory potential use for bio-sustainable industrial scale up (orange, bottom)

In this work we presented an alternative, bio-sustainable and cost-effective approach to produce ω-6 and ω-3 PUFAs using the single cell eukaryote *C. fasciculata* (Fig. 7). *C. fasciculata* are formidable producers of a very wide variety of FAs, which are synthesised via a vast repertoire of FAs desaturases and elongases [28]. Taking advantage of this biochemical feature, as well as of *C. fasciculata*’s adaptability, plasticity and rapid growth in cheap media at ambient temperature, we bioengineered the FA metabolism using genetic manipulation, chemical supplementation and temperature variation (Fig. 7) [28]. Thus, we were able to successfully build a low-cost and high-yielding ω-6 and ω-3 PUFAs cell-factory (Fig. 7). We showed that, by overexpressing the endogenous desaturases Cf-Δ6 or Cf-Δ4 in conjunction with the elongase Cf-Elo4, the level of ω-6 and ω-3 PUFAs present in cell biomass increases significantly. Particularly, the cell-factories were able to produce larger amounts of ω3–18:3 (α-linolenic acid), ω3–22:5 (docosapentaenoic acid), ω6–22:4 (adrenic acid) and ω6–20:4 (arachidonic acid), which are among the most requested PUFAs in the food and nutraceutical industries (Fig. 7) [34]. Our cell-factories produced even higher level of this ω-6 and ω-3 PUFAs, when the culturing temperature was lowered from 27˚C to 20˚C. This highlights that, to efficiently increase ω-6 and ω-3 PUFAs production, it is essential that one of the desaturases and the elongase are overexpressed simultaneously. This is key for the cells to maintain the correct membrane fluidity [30]. In fact, larger amounts of VLC-PUFAs, which have long and ‘kinked’ acyl-chains, are necessary to reduce the degree of packing in the lipid bilayer, caused by the temperature reduction, and/or by the change in the ratio S/U FAs as consequence of the upregulation of Cf-Δ6, Cf-Δ4 and Cf-Elo4. This ensures that the appropriate membrane fluidity is maintained to allow the plethora of cellular process involving membrane biogenesis and fusion to continue [35–37]. We were also able to push the *C. fasciculata* cell-factories one step further, by growing the cells on cheap and commercial cooking oil, such as coconut oil and used sunflower oil [28]. We confirmed that the cell factories efficiently uptake and remodel FA sources from these oils to make even higher amounts of ω-6 and ω-3 PUFAs. Upon these conditions, the OE-D4-Elo4 cells were the most efficient cell-factories. Particularly, from a 20 mL culture we obtained 1 g of wet biomass, which yielded ~ 250 mg of dry mass of which ~ 22.5 mg were lipids, containing large amounts of FAs, of which 28% (in used sunflower oil) and 51% (in coconut oil) are ω-6 and ω-3 PUFAs, relative to the WT (23% and 38% for the two oils respectively) (Fig. 7). Comparing these yields with those of vegetable oil plants, the advantage is evident: plants, if not genetically modified, and with very high economic and environmental costs, make less than 1% of 18:3 and cannot produce any ω-3-22:5 [16]. On the other hand, despite the production of ω-3-VLC-PUFAs by algae (i.e. *Euglena gracilis*, *Pavlova lutheri* etc.) typical reaches ~ 18 mg/g (dry weight) scale [38, 39], which is at similar or slightly below that of our cell factories, the procedures and the conditions to achieve these yields (optimal temperature, light, pH, type of water, humidity, expensive carbon sources etc.) have very high production costs [16]. We believe that our promising cell-factories may offer a valid approach to respond to the current world-wide shortage of dietary ω-3 and ω-6 PUFAs, which is mainly caused and continuously worsened by the agricultural and industrial progress and by climate change [11, 12]. Particularly, this situation has dramatically affected the marine ecosystem, which is currently the main source of dietary ω-3 and ω-6 PUFAs [12, 40]. The use of vegetable oil plants, which are currently another highly exploited FA sources, does not meet the current increasing demand of PUFAs either [16, 41]. Furthermore, the industrial chemical synthesis of FAs is also very challenging, time consuming and certainly non-environmentally friendly [17]. On the other hand, the latest biotechnological advances for the production of dietary ω-3 and ω-6 PUFAs are often too expensive [42, 43]. Thus, this *C. fasciculata* PUFAs cell-factory can represent an innovative and alternative bioengineered microbial platform to access ω-3 and ω-6 PUFAs in a larger amount, and most importantly, adopting a sustainable and low-cost approach (Fig. 7). *C. fasciculata* PUFAs cell factories have all the desirable characteristics, such as fast growth, large biomass and cheap medium, for an easily achievable scale up to a multi-kg scale in an industrial setting using suitable/simple bioreactors. This process has low costs and a very low environmental impact: just some aeration, non-expensive, nor particular culturing conditions, such as UV light or CO_2_ are required to grow the cells, but rather cheap media (overall cost of the media to obtain 1 g of wet biomass ~ £0.90 at lab scale and without industrial optimisation), including valorised biowaste as media supplement (Fig. 7) [44, 45]. It is possible to predict that such a simple system will have very little limitation and or challenges in terms of industrial scale up. Especially when considering that we are able to yield at lab scale large quantities of ω-3 and ω-6 PUFAs-enriched cell biomass, which could go through industrial downstream processes (i.e. lyophilisation) to obtain the PUFA enriched final products, ready to be used, avoiding challenging purification steps (Fig. 7) [46]. These enriched products could find applications as cheap and highly valuable animal and soil fortified feedstocks allowing for larger quantities of ω-3 and ω-6 PUFAs to enter the food chain (Fig. 7) [46]. The food waste that is eventually generated from such process could also be recycled and used as ‘renewable’ sources for the cell-factories to continue the production of ω-3 and ω-6 PUFAs (Fig. 7) [44, 45]. This platform has great potential for development of an alternative and novel microbial system to respond to the increasing world demand of PUFAs, following a bio sustainable process inspired by the concept of circular economy (Fig. 7) [45, 47].

## Materials and methods

Unless otherwise stated, all reagents and materials were purchased from Sigma, Promega, New England Bio Labs (NEB) or VWR.

### Cell culture

*C. fasciculata* clone HS6 parasites were grown at pH 7.6 in buffered serum-free medium containing 5 g/L yeast extract, 4 g/L tryptone, with or without 15 g/L or 1.5 g/L sucrose, 4.4 g/L triethanolamine hydrochloride, with or without 0.5% v/v Tween-80, 10 µg/mL haemin and 71.5 g/L HEPES. Cells were incubated at 27 °C–20 °C with gentle agitation, maintained at mid-log phase and passaged every 3 days [48]. Cell counting was performed using a haemocytometer.

### Chemical supplementation of *C. fasciculata* culture media with commercially available oil sources

20 mL (the volume of media for each cell culture sample) of buffered serum-free minimal media with 10% Tween-80 and 10% sucrose were supplemented with 150 µL of coconut oil or 150 µL of used sunflower, and cultured as above [28].

### Growth curves

*C. fasciculata* clone HS6 cells were grown to mid-log phase in standard serum-free media, or in minimal media with 10% of Tween-80, and with 10% of sucrose, with or without oil supplementation, and distributed into non-vented flasks at an equal density of 5 × 10^4^ cell/mL in the presence or absence of the appropriate concentration of selection drugs, where required according to the cell line. The cells were grown for 48–72 h and counted using a haemocytometer every 24 h.

### Generation of the overexpression vector pNUS-ss-MCS-C-term-V5-Hyg

The vector pNUS-ss-MCS-C-term-V5-Hyg was modified from the vector pNUS-SP-N-term-Hyg-C-term-His [29]. A synthetic G-block oligonucleotide containing 5’-*Nde*I and 3’-*EcoR*I restriction sites, a multiple cloning sites region (MCS) including *Bg*III, *Not*I and *EcoR*V restriction sites, a secretory signal sequence (ASKISRLLLAALLVAAAITADAL), and a C-terminal sequence encoding for a V5-tag (GKPIPNPLLGLDST) was synthesised by Integrated DNA Technologies (IDT). The G-block was PCR amplified using KOD Hot Start DNA Polymerase (Novagen) using primers 5’-TTT ATA TC*C ATA TG*G CCT CTA AGA TCA GTC GTC GT-3’ and 5’-AAA TGT *GAA TTC* TTT TTA AGA CAG GCC CAG AAG-3’, containing *Nde*I and *EcoRI* restriction sites (underlined regions). The PCR product (152 bp) was purified by a column clean up kit (Qiagen), and this and the vector were subsequently digested with *Nde*I and *EcoR*I and then ligated using T4 ligase (Thermo Fisher). The plasmid obtained was defined as pNUS-ss-MCS-C-term-V5-Hyg and the presence of the insert was confirmed by PCR using GoTaq DNA polymerase (Promega) (expected size product 714 bp), and sequencing at Eurofins Genomic, using the following forward 5’- TGT AAA ACG ACG GCC AGT G -3’ and reverse 5’- GCG AGT CACCGT GCA GC -3’ primers.

### Generation of the overexpression vector pNUS-ss-MCS-C-term-V5-Neo

The neomycin resistance cassette gene was amplified using KOD Hot Start DNA Polymerase (Novagen) the following forward and reverse primers 5’- AAA AA*G TCG AC*A TGA TTG AAC AAG ATG GAT TGC − 3’ and 5’- AAA AA*G GAT CC*T CAG AAG AAC TCG TCA AGA AGG − 3’ containing *Sal*I and *BamH*I restriction sites (underlined). The purified product (818 bp) was purified and digested with *Sal*I and *BamH*I and ligated into the appropriately digested pNUS-ss-MCS-C-term-V5-Hyg vector. The plasmid obtained was defined as pNUS-ss-MCS-C-term-V5-Neo and the presence of the insert was confirmed by PCR using GoTaq DNA polymerase (Promega) (expected size product 818 bp), and sequencing at Eurofins Genomic, using the following forward 5’- TCA AGC GTC GAC ATG ATT GAA C -3’ and reverse 5’- CTG CTG GAT CCT CAG AAG − 3’ primers.

### Generation of the *C. fasciculata* Δ6-desaturase overexpression construct using pNUS-ss-MCS-C-term-V5-Hyg vector

The ORF of the putative *C. fasciculata* Δ6-desaturase (CFAC1_280079900.1) was amplified from *C. fasciculata* HS6 clone genomic DNA using KOD Hot Start DNA Polymerase (Novagen) and using the following forward and reverse primers 5’- GGC TTC AGA TCT ATG GTC TTC GAG CTC ACC − 3’ and 5’- TTT TTT GC G GCC GCA TGC TGC TTC TTC TC -3’, containing *Bgl*II and *Not*I restriction sites (underlined). The purified product (1221 bp) was digested with *Bgl*II and *Not*I and ligated into appropriately digested pNUS-ss-MCS-C-term-V5-Hyg vector. The plasmid obtained was defined as pNUS-ss-Cf-Δ6-C-term-V5-Hyg and confirmed by PCR using GoTaq DNA polymerase (Promega) (expected size product 2018 bp), and sequencing at Eurofins Genomic, using the following forward 5’-TGT AAA ACG ACG GCC AGT G-3’ and reverse 5’-GCG AGT CACCGT GCA GC-3’ primers.

### Generation of the *C. fasciculata* Δ4-desaturase overexpression construct using pNUS-ss-MCS-C-term-V5-Hyg vector

The ORF of the putative *C. fasciculata* Δ4-desaturase (CFAC1_110026100.1) was amplified including 5’-UTR and 3’-UTR regions respectively upstream and downstream of the target gene from *C. fasciculata* HS6 clone genomic DNA, using KOD Hot Start DNA Polymerase (Novagen) and the following forward and reverse primers 5’-AAA AA *GGATCC* CTTCTATGGGCTTCTTGAGACAGC-3’ and 5’-AAA AAG TCG ACG ATT CAC GCG TCT TGT TCG CTC TTA TA-3’, containing *BamH*I and *Sal*I restriction sites (underlined). The product (2397 bp) was purified by a column clean up kit (Qiagen) and this and the vector were subsequently digested with *BamH*I and *Sal*I. The product was ligated into the pET15b-linker vector using T4 ligase (Thermo Fisher). The plasmid obtained was defined pET15b-5’-UTR-Cf-Δ4 − 3’-UTR and the presence of the insert was confirmed by PCR using GoTaq DNA polymerase (Promega), and sequencing at Eurofins Genomic, using the following the following forward T7-1 primer 5’-AATACGACTCACTATAGGG-3’ and reverse pET-RP2 primer 5’-TAGTTATTGCTCAGCGGTG-3’. The plasmid pET15b-5’-UTR-Cf-Δ4 − 3’-UTR was used as a template to amplify the ORF of Cf-Δ4, using KOD Hot Start DNA Polymerase (Novagen) using the following forward and reverse primers 5-TTT TT A GAT CTA TGG CCC CCG C-3’ and 5’-TTT TTG ATA TCC TGC GTC TTC AC-3’, containing *Bgl*II and *EcoR*V restriction sites (underlined). The product was purified by a column clean up kit (Qiagen) and this and the vector were subsequently digested with *Bgl*III and *EcoR*V. The product was ligated into the pNUS-ss-MCS-C-term-V5-Hyg vector using T4 ligase. The plasmid obtained was defined as pNUS-ss-Cf-Δ4-C-term-V5-Hyg and the presence of the insert was confirmed by PCR, using GoTaq DNA polymerase (Promega) (expected size product 1941 bp), and sequencing at Eurofins Genomics, using the following forward 5’- TCA AGC GTC GAC ATG ATT GAA C -3’ and reverse 5’- CTG CTG GAT CCT CAG AAG − 3’ primers.

### Generation of the *C. fasciculata* Elo4-elongase overexpression construct using pNUS-ss-MCS-C-term-V5-Neo vector

The ORF of the putative *C. fasciculata* Elo4 elongase (CFAC1_110016500.1) gene was amplified including 5’-UTR and 3’-UTR regions respectively upstream and downstream of the target gene from *C. fasciculata* HS6 clone genomic DNA using KOD Hot Start DNA Polymerase (Novagen, aand the following forward and reverse primers 5’- TTT TTG GAT CCA GAG TGA GAG AGT CCT ACA C -3’ and 5’- TTT TT*G TCG AC*C GCG TTT GTG TAT ATG CGT G -3’, containing *BamH*I and *Sal*I restriction sites (underlined).The product (1507 bp) was purified by a column clean up kit (Qiagen) and this and the vector were subsequently digested with *BamH*I and *Sal*I. The product was ligated into the pET15b-linker vector using T4 ligase (Thermo Fisher). The plasmid obtained was defined as pET15b-5’-UTR-Cf-Elo4-3’-UTR and the presence of the insert was confirmed by PCR using GoTaq DNA polymerase (Promega) (expected size product 1713 bp), and sequencing at Eurofins Genomics using the following forward T7-1 primer 5’-AATACGACTCACTATAGGG-3’ and reverse pET-RP2 primer 5’-TAGTTATTGCTCAGCGGTG-3’. The plasmid pET15b-5’-UTR-Cf-Elo4-3’-UTR was used as a template to amplify Cf-Elo4 ORF using KOD Hot Start DNA Polymerase (Novagen) and the following forward and reverse primers 5’- TTT TTA GAT CTA TGT CGC ACC CGG CCA TTC − 3’ and 5’- TTT TTG CGG CCG CCT TTT TCT TCT TG -3’, containing *Bgl*II and *Not*I restriction sites (underlined).The product was purified by a column clean up kit (Qiagen) and this and the vector were subsequently digested with *Bgl*III and *Not*I. The product was ligated into the pNUS-ss-MCS-C-term-V5-Neo vector using T4 ligase (Thermo Fisher). The plasmid obtained was defined as pNUS-ss-Cf-Elo4-C-term-V5-Neo and the presence of the insert was confirmed by PCR using GoTaq DNA polymerase (Promega) (expected size product 1652 bp), and sequencing at Eurofins Genomics, using the following forward 5’- TCA AGC GTC GAC ATG ATT GAA C -3’ and reverse 5’- CTG CTG GAT CCT CAG AAG − 3’ primers.

### Transfection of *C. fasciculata*

1 × 10^7^*C. fasciculata* mid-log phase parasites were harvested at 800 x g for 10 min and transfected using Lonza T-cell nucleofector kit and Amaxa-electroporator using program X-014. The transfected parasites were transferred into a flask containing 5 mL of serum-free buffered media and incubated at 27°C with gentle agitation for 24 h. Flasks for wild type and a drug free control were also included. After 24 h, the transfected cells were supplemented with 6.25 µg/mL of hygromycin or neomycin, as appropriate, and to the WT control. The cells were passaged every three days, with doubling of the drug concentration at each passage to finally reach 200 µg/mL of either hygromycin or neomycin, as appropriate. The cells that showed the highest growth rate were selected. OE-Δ6, OE-Δ4, OE-Elo4, OE-Δ6-Elo4 and OE-Δ4-Elo4 genotypes were verified by PCR analysis of plasmid DNA isolated from parental and overexpression cell lines, using GoTaq DNA polymerase (Promega), and the following forward 5’- GGC TTC AGA TCT ATG GTC TTC GAG CTC ACC − 3’ (for Cf-Δ6), 5-TTT TT A GAT CTA TGG CCC CCG C-3’ (for Cf-Δ4) or 5’- TTT TT*A GAT CT*A TGT CGC ACC CGG CCA TTC − 3’ (for Elo4) and reverse primers 5’- GGCTTGGCTGGAGCTAGTGGAGG-3’.

### Lipid extraction

2 × 10^7^ cells of mid log phase *C. fasciculata* were collected by centrifugation at 800 x *g* for 10 min at room temperature. The cell pellet was washed with phosphate-buffered saline (PBS), then re-suspended in 100 µL of PBS and transferred to a glass vial. 375 µL 2:1 (v/v) of MeOH: CHCl_3_ solution was added for biphasic separation based on the following method described by Bligh-Dyer [49]. The samples were vigorously agitated at 4 °C overnight. The internal standard D_27_-myristic acid (250 pmol) were added for GC-MS samples from genetically manipulated *C. fasciculata* supplemented with oils, where required. The samples were made biphasic by the addition of 125 µL CHCl_3_ and 125 µL distilled sterile water, followed by vigorous agitation after each addition. The samples were centrifuged at 1000 x *g* for 5 min to allow the aqueous and the organic layers to separate. The organic phase was transferred into a new glass vial and dried under nitrogen gas stream to obtain the total lipid extract ready to use and stored at 4 °C until further analysis.

### Fatty acids transmethylation and gas chromatography – Mass spectroscopy (GC-MS) samples preparation

Methylation of fatty acids to form fatty acid methyl esters (FAMEs) was performed on the dry total lipid extracts. The reaction (total volume 1 mL) was conducted in a glass vial [50]. 100 µL of toluene were added, followed by 750 µL of MeOH and 150 µL of 8% HCl in 85:15 (v/v) MeOH: H_2_O solution to allow the esterification of the free fatty acids. The reaction was left to react at 45 °C overnight. Upon drying under a nitrogen gas stream, the FAMEs were extracted with a 1:1 (v/v) hexane: H_2_O solution. The FAME extracts were dried under a nitrogen gas stream. The FAME extracts were dissolved in dichloromethane, typically 15 µL, of which 3 µL were analysed by GC-MS on an Agilent Technologies GC-6890 N gas chromatograph coupled to an MS detector-5973. Separation by GC was performed using a Phenomenex ZB-5 column (30 m x 25 mm x 25 mm), with a temperature program of 70 °C for 10 min, followed by a gradient to 220 °C, at 5 °C/min, which was maintained at 220 °C for a further 15 min. Mass spectra were acquired from 50 to 500 amu. FAME species were assigned by comparison of the retention time, fragmentation pattern, use of FAME standards, and the online FAMEs mass spectrometry database (http://www.lipidhome.co.uk/ms/methesters/me-arch/index.htm).

### Western blot probed with an anti-V5 antibody and samples preparation

1 × 10^7^ mid-log-phase *C. fasciculata* parasites were harvested by centrifugation at 800 x g for 10 min. The cells were washed in PBS twice and re-suspended in 50 µL of 2 x SDS sample buffer and incubated at 65 °C for 10 min. SDS-PAGE was performed on 12% acrylamide gel. The protein gel was then transferred onto a membrane (GE Healthcare Life Science UK) and the membrane blocked in 5% semi skimmed milk powder in PBS. The membrane was incubated for 1 h at room temperature with Odyssey blocking buffer and 0.2% PBS-T containing mouse anti-V5 primary antibody (kindly given by Dr Dan Young and Prof Rick Randall, University of St Andrews) at 1:5,000 dilution. The membrane was washed for 3 × 10 min with 0.2% PBS-T buffer. Subsequently, the membrane was incubated for 1 h at room temperature with secondary goat anti-mouse conjugated with DyLight 800 CW antibody at 1:15,000 dilution, and washed as above, before the fluorescence was detected with Odyssey western blot detection system (Licor Odyssey).

### Immunofluorescence microscopy for *C. fasciculata*

1 × 10^6^*C. fasciculata* parasites were harvested at 800 x g for 10 min and washed with PBS. The cells were washed with PBS and resuspended in 100 µL of 4% paraformaldehyde in PBS and incubated at room temperature for 15 min. After this time, the cells were spun at 3000 rpm for 2 min, the supernatant removed, and the cells washed with PBS and resuspended in 100 µL fresh PBS. 50 µL of cells were added to Polysine^®^ slides (VWR 631 − 0107) and allowed to adhere overnight at room temperature in a sealed container on a damp tissue to prevent evaporation. The cells were re-hydrated with 100 µL of dH_2_O for 5 min and washed with 100 µL of PBS for 2 × 5 min using a pipette. The cells on the slide were incubated with 100 mM glycine in PBS for 5 min. The cells were washed for 3 × 5 min with 100 µL of PBS. At this point 50 µL of 0.1% TritonX-100 solution in PBS were added to permeabilise the cells for 10 min. The cells were washed for 3 × 5 min with 100 µL of PBS and blocked for 20 min with 1% bovine serum albumin (BSA) in PBS. The cells were washed for 1 × 5 min with 100 µL of PBS. (i) For V5-tag localisation, the primary mouse anti-V5 primary antibody was diluted to 1:500 in 1% BSA-PBS, and the cells were incubated with this solution at room temperature for 2 h. The cells were washed with 100 µL of 1% BSA-PBS for 3 × 5 min. The secondary horse anti-mouse Texas-Red antibody (Vector Laboratories) was diluted to 1:1,000 in 1% BSA-PBS, and the cells were incubated with this solution at room temperature for 2 h. The slides were washed for 3 × 5 min with PBS in a Coplin jar. The slides were thoroughly dried with the use of a pipette. (ii) To stain the mitochondrial membrane with MitoTracker Green^®^ the cells were incubated with 50 µL of MitoTracker Green^®^ (2 ng/mL in PBS) for 20 min in the dark. (iii) To stain the DNA, the cells were incubated with 50 µL 4,6-diamidino-2-phenylindole (DAPI) (2 µg/mL, in PBS) for 5 min in the dark. The slides were washed for 3 × 5 min with PBS. A drop of antifade agent was added to each sample on the slide, covered with a coverslip and let dry at room temperature overnight, before sealing the coverslips with nail polish. Images were acquired with a fluorescence confocal microscope (DeltaVision Imaging System), connected to a digital camera system, and processed by ImageJ image analysis software.

### Statistical analysis

For all growth curves and fatty acids experiments values are the mean of three independent biological replicates (*n* = 3). Error bars represent the standard deviation of each mean calculated by GraphPad PRISM 6.0. Statistical analysis, when required, was also performed by GraphPad PRISM 6.0 using One-way or 2-ways ANOVA multiple comparisons or based on a Tukey or Dunnet t-test with a 95% confidence interval.

## Electronic supplementary material

Below is the link to the electronic supplementary material.

Supplementary Material 1

Supplementary Material 2

Supplementary Material 3

## Data Availability

No datasets were generated or analysed during the current study.

## Abbreviations

BSA: Bovine serum albumin
DAPI 14: 4,6-diamidino-2-phenylindole, 14
EPUFA(s): Essential polyunsaturated fatty acid(s), 2
ER: Endoplasmic reticulum, 4
FA(s): Fatty acid(s), 1
FAMEs: Fatty acid methyl esters, 13
GC-MS: Gas chromatography – Mass spectroscopy, 13
IDT: Integrated DNA Technologies, 10
MCS: Multiple cloning sites, 3
PBS: Phosphate-buffered saline, 13
PUFA(s): Polyunsaturated fatty acid(s), 1
SAFA(s): Saturated fatty acid(s), 2
UFA(s): Unsaturated fatty acid(s), 2
VLC-PUFA(s): Very long chain polyunsaturated fatty acid(s), 5
