# Supplementary material for: Building a cell-factory in Crithidia fasciculata: a bio-sustainable system to produce high-value polyunsaturated fatty acids
Source: Microb Cell Fact. 2025 Jun 23;24:142. doi: 10.1186/s12934-025-02760-7 (PMC12183914; doi:10.1186/s12934-025-02760-7)
Supplement: Supplementary file 3 — Supplementary Material 3 [file 12934_2025_2760_MOESM3_ESM.docx]

Supplementary information – Figure legends

A


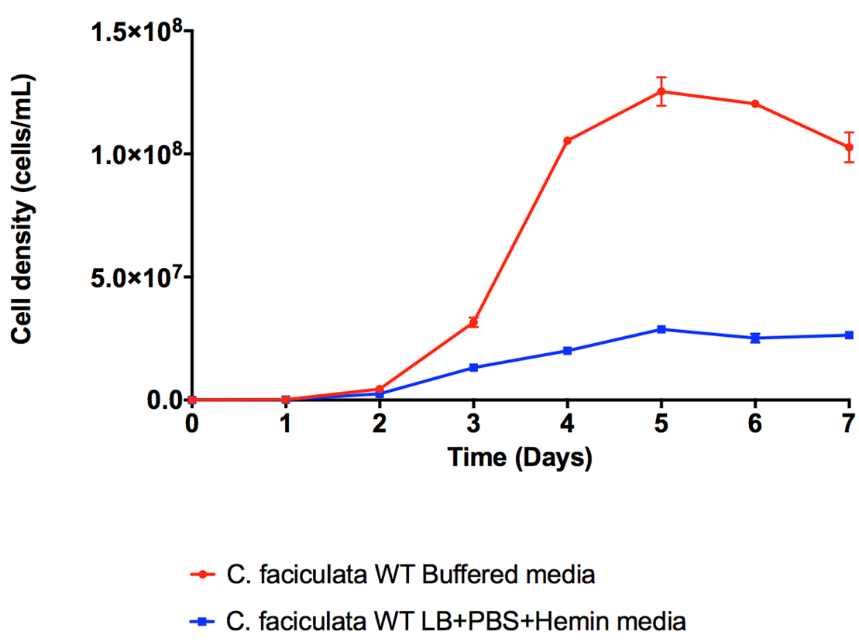


B


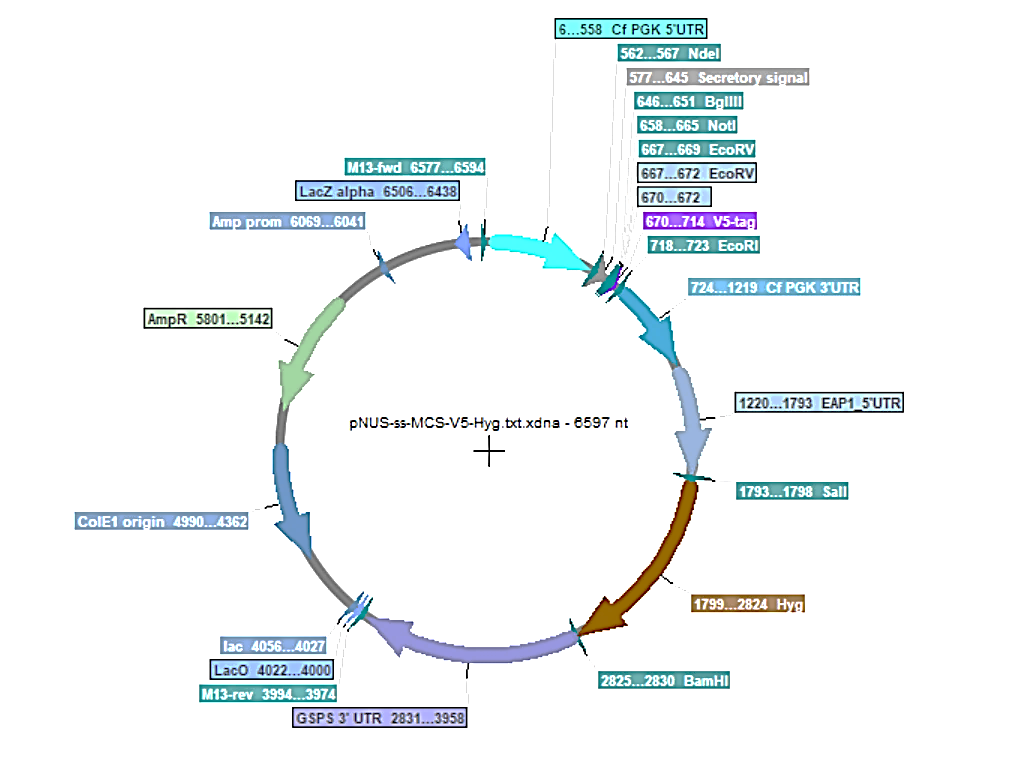


Figure S1. A) Growth curves of *C. fasciculata* WT cultured in serum-free buffered media and in Luria-Bertani broth. The graph represents the growth curves over 7 days of *C. fasciculata* WT cultured in standard media (in serum-free buffered media) at 27˚C (red line) and in Luria-Bertani (LB) broth (blue line). Values are the mean of three independent biological replicates (n=3). B) The vector map of pNUS-ss-MCS-C-term-V5-Hyg. The figure is a schematic representation of pNUS-ss-MCS-C-term-V5-Hyg obtained using Snap gene molecular biology software.


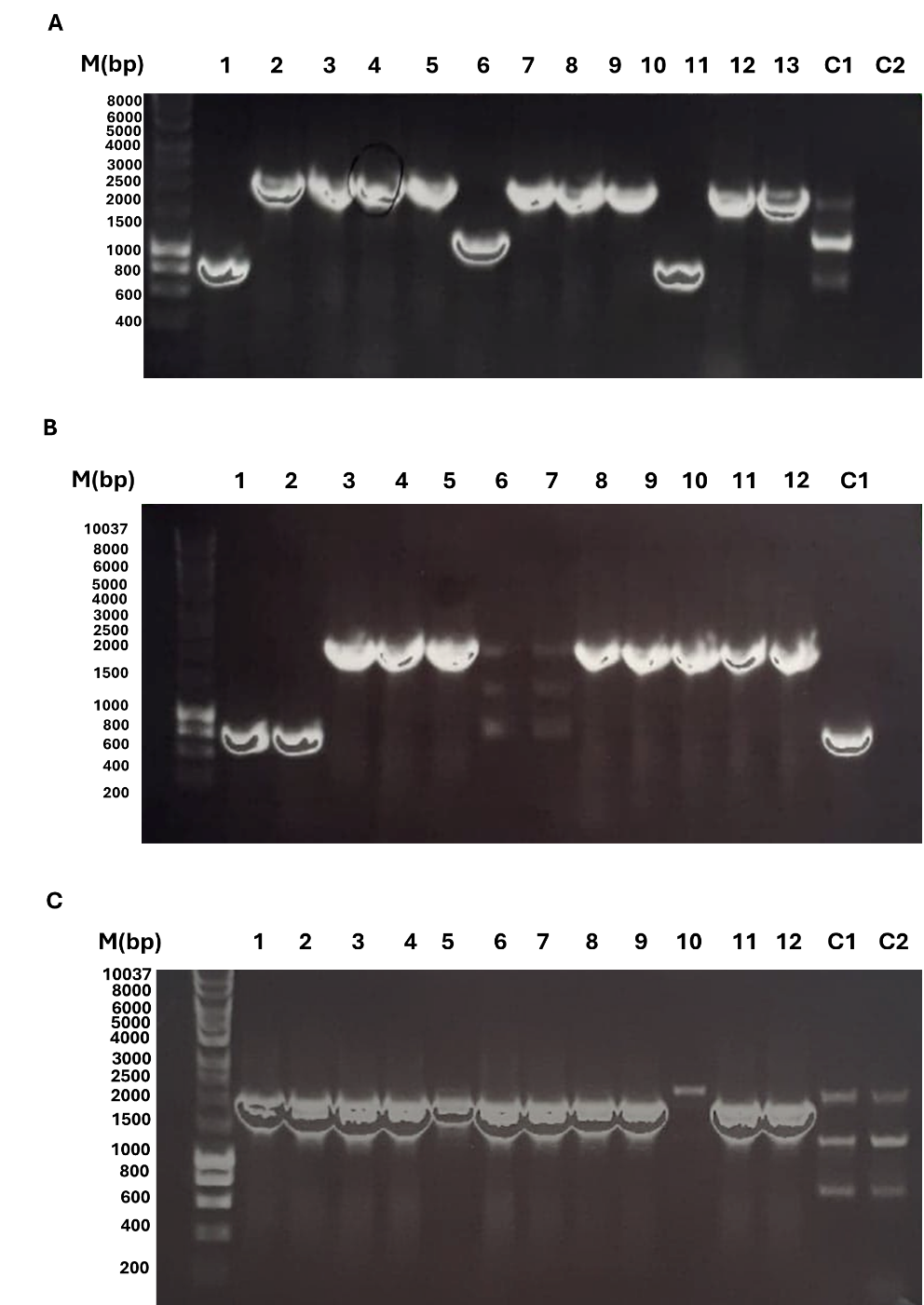


Figure S2. PCR colony screening A) Confirming the gene encoding Cf-Δ6 cloned into pNUS-ss-MCS-C-term-V5 vector. Lanes 1-13 are a PCR amplification of the gene encoding Cf-Δ6 in the pNUS-ss-Cf-Δ6-C-term-V5-Hyg plasmid from *E. coli* single colonies, compared to *E. coli* single colonies containing empty vector (C1) and no ligase (C2). The product (lanes 2-5, 7-10 and 12-13) is an intense band at around 2000 bp (expected size 2018 bp). B) confirming the gene encoding Cf-Δ4 cloning into pNUS-ss-MCS-C-term-V5-Hyg vector. Lanes 1-12 are a PCR amplification of the gene encoding Cf-Δ4 in the pNUS-ss-Cf-Δ4-C-term-V5-Hyg plasmid from *E. coli* single colonies, compared to *E. coli* single colonies containing empty vector (C1) and no ligase (C2). The product (lanes 3-5 and 8-12) is an intense band at around 2000 bp (expected size 1941 bp). C) confirming the gene encoding Cf-Elo4 cloning into pNUS-ss-MCS-C-term-V5-Neo vector. Lanes 1-12 are a PCR amplification of the gene encoding Cf-Elo4 in the pNUS-ss-Cf-Elo4-C-term-V5-Neo plasmid from *E. coli* single colonies, compared to *E. coli* single colonies containing empty vector (C1) and no ligase (C2). The product (lanes 3-5 and 8-12) is an intense band at just above 1500 bp (expected size 1652 bp).


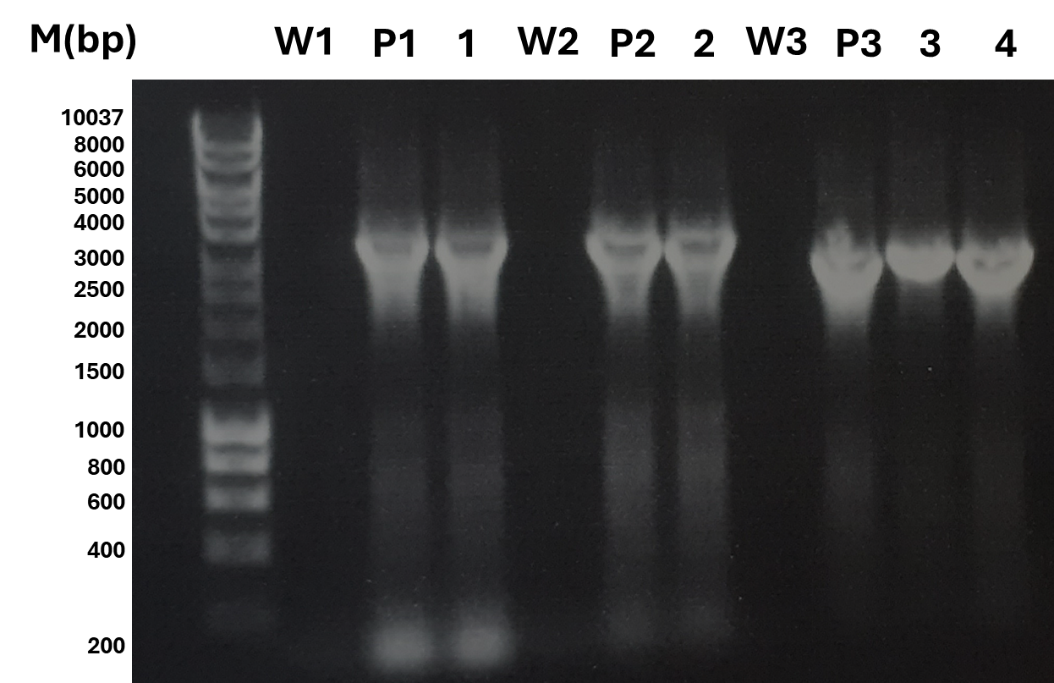


Figure S3. PCR amplification confirms the integration of plasmid DNA encoding Cf-Δ6, Cf-Δ4 and Cf-Elo4 in *C. fasciculata*.  Lanes W1, P1 and 1 are a PCR amplification of pNUS-ss-Cf-Δ6-C-term-V5-Hyg using primers targeting Cf-Δ6 and Hyg, where W1 is WT negative control, P1 is the plasmid positive control, and 1 is the plasmid extracted from OE-D6-ELO4. The product is a bright band between 3000-4000 bp (expected size 3469 bp) present in P1, absent in W1 and integrated in OE-D6-Elo4 (lane 1). Lanes W2, P2 and 2 are a PCR amplification of pNUS-ss-Cf-Δ4-C-term-V5-Hyg using primers targeting Cf-Δ4 and Hyg, where W2 is WT negative control, P2 is the plasmid positive control, and 2 is the plasmid extracted from OE-D4-ELO4. The product is a bright band between 3000-4000 bp (expected size 3410 bp) present in P2, absent in the W2 and integrated in OE-D4-ELO4 (lane 2). Lanes W3, P3, 3 and 4 are a PCR amplification of pNUS-ss-Cf-Elo4-C-term-V5-Neo using primers targeting Cf-Elo4 and Neo, where W3 is WT negative control, P3 is the plasmid positive control, and 3 and 4 are the plasmid extracted from OE-D6-ELO4 and OE-D4-ELO4, respectively. The product is a bright band between 2500-3000 bp (expected size 2952 bp) present in P3, absent in the W3 and integrated in OE-D6-ELO4 (lane 3) and OE-D4-Elo4 (lane 4).


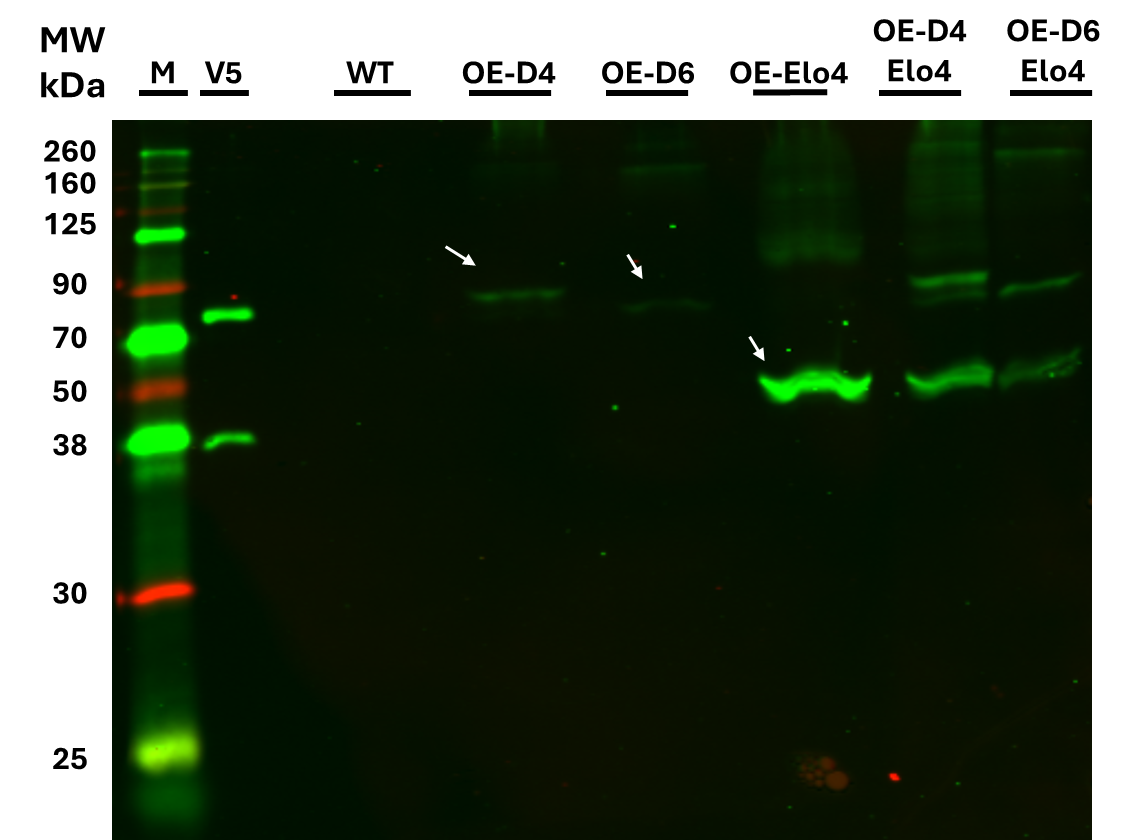


Figure S4. Western blot, probed with an anti-V5 tag antibody, confirms the overexpression of Cf-Δ6, Cf-Δ4 and Cf-Elo4 in *C. fasciculata*.  The figure represents the western blot, probed with an anti-V5 tag antibody, of the protein extract from *C. fasciculata* WT control and *C. fasciculata* Cf-Δ6 (OE-D6), Cf-Δ4 (OE-Δ4) and Cf-Elo4 (OE-Elo4) overexpression, and the *C. fasciculata* cell-factories overexpressing Cf-Δ6 or Cf-Δ4 desaturases in conjunction with Cf-Elo4 elongase (OE-D6-ELO4 and OE-D4-ELO4). The cells were grown for 48 h and harvested at 1 x 10^7^ cell/mL. The green, fluorescent signals for the protein bands highlight the confirmed overexpression of Cf-Δ6 (expected size 48 kDa, of Cf-Δ4 (expected size 49 kDa) and Cf-Elo4 (expected size 35 kDa). V5, is the positive control, showing V5 tagged proteins (first column) obtained from the cell lysate.


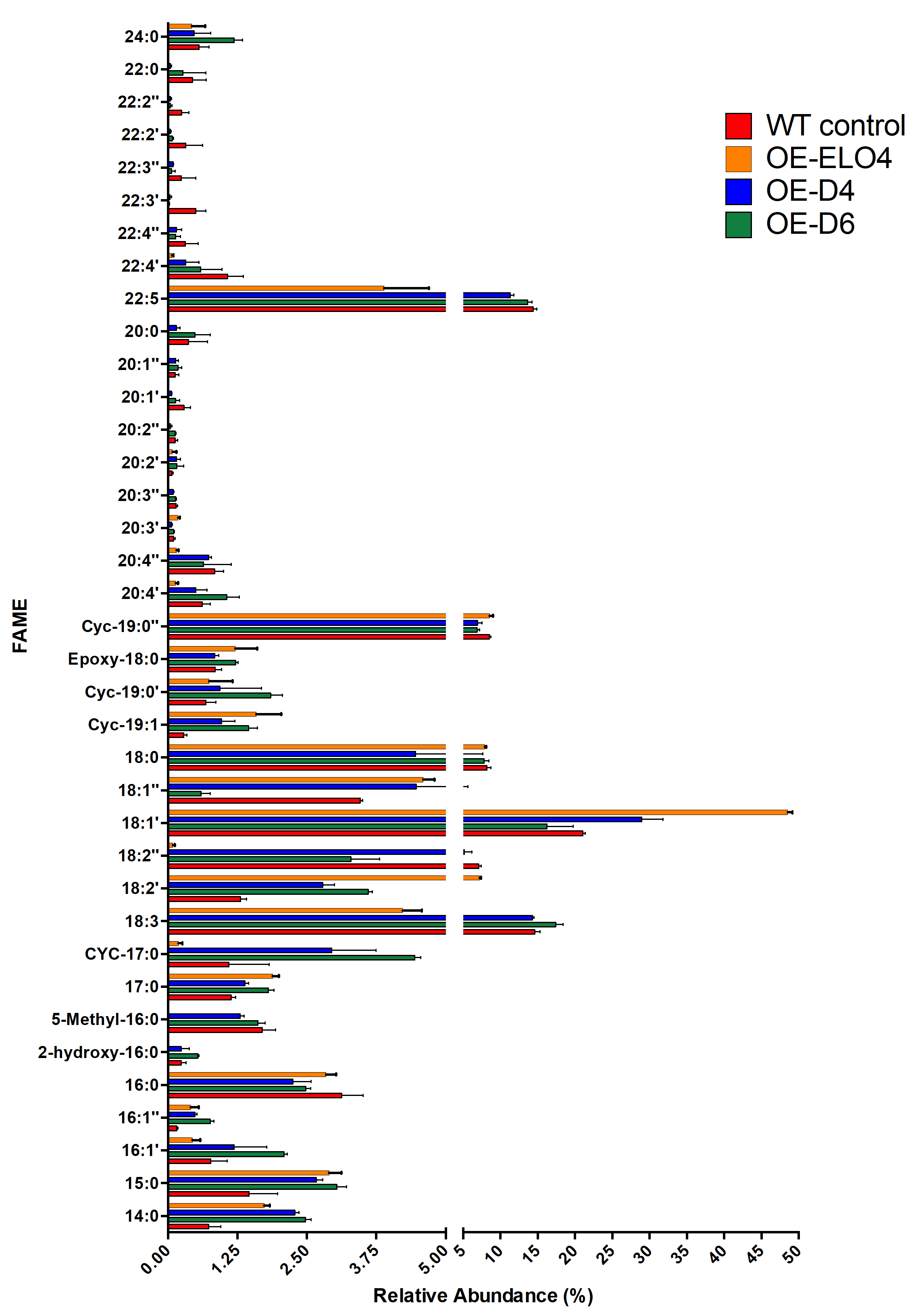


Figure S5. Fatty acid profile of genetically manipulated *C. fasciculata* cells overexpressing Cf-Δ6 or Cf-Δ4 or Cf-Elo4 and grown in standard media at 27˚C.  The bar chart shows the FAs (Y axis, the order follows increasing retention time) and the relative abundance (X axis) found in *C. fasciculata* overexpressing Cf-Δ6 (OE-D6) or Cf-Δ4 (OE-D4) or Cf-Elo4 (OE-ELO4) and WT control, grown in standard media at 27˚C, as shown in the legend. Values are the mean of three independent biological replicates (n=3). Error bars represent the standard deviation of each mean (±). All FAs were identified using GC-MS based upon retention time, fragmentation, and comparison with standards. Statistical analysis was performed by PRISM 6 using One-way ANOVA multiple comparisons based on a Tukey t-test with a 95% confidence interval.


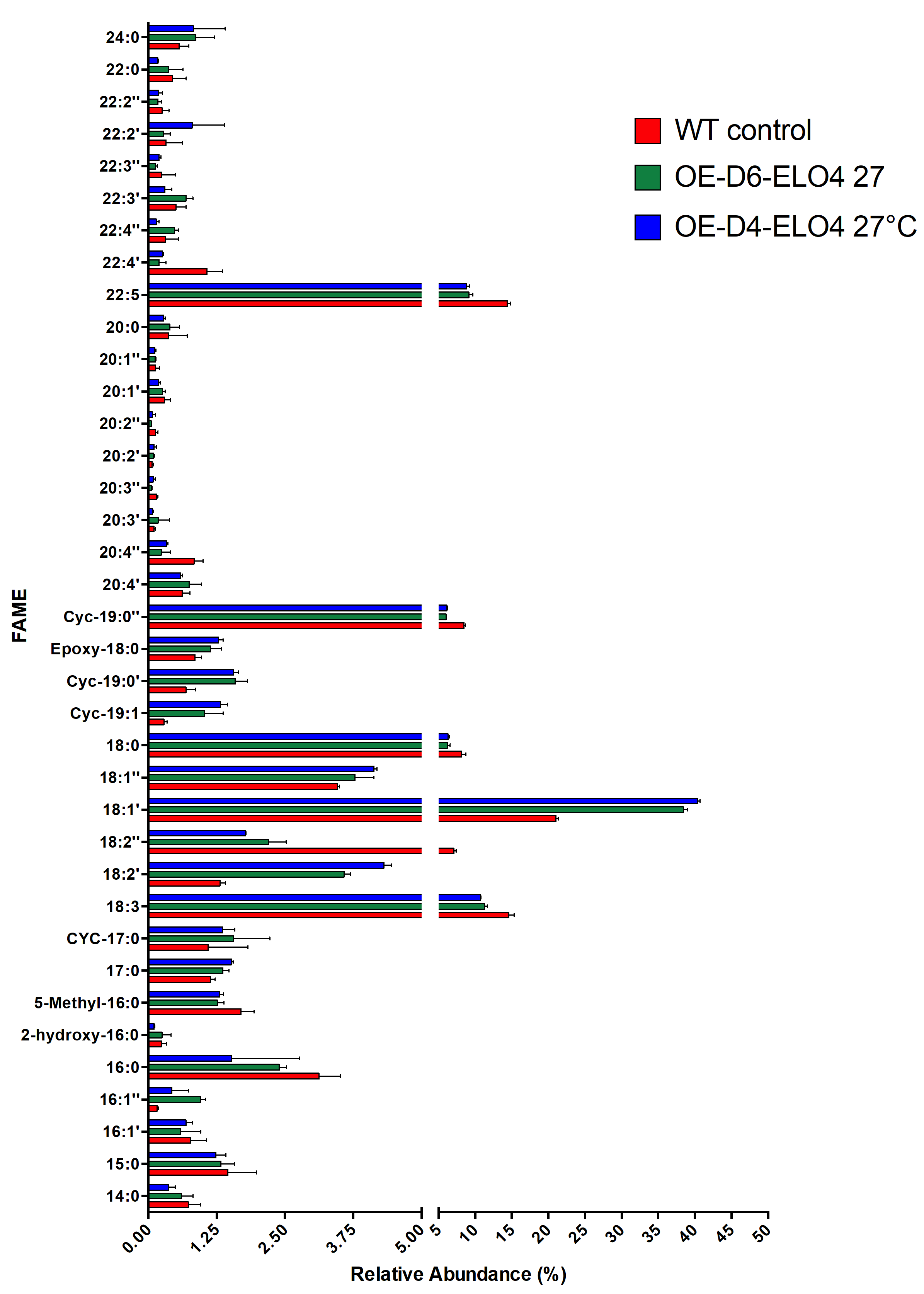


Figure S6. Fatty acid profile of genetically manipulated *C. fasciculata* cells overexpressing Cf-Δ6 or Cf-Δ4 in conjunction with Cf-Elo4 grown in standard media at 27˚C. The bar chart shows the FAs (Y axis, the order follows increasing retention time) and the relative abundance (X axis) found in *C. fasciculata* overexpressing Cf-Δ6 or Cf-Δ4 in conjunction with Cf-Elo4 (OE-D6-Elo4 and OE-D4-Elo4) and WT control, grown in standard media at 27˚C. Values are the mean of three independent biological replicates (n=3). Error bars represent the standard deviation of each mean (±). All FAs were identified using GC-MS based upon retention time, fragmentation, and comparison with standards. Statistical analysis was performed by PRISM 6 using One-way ANOVA multiple comparisons based on a Tukey t-test with a 95% confidence interval.


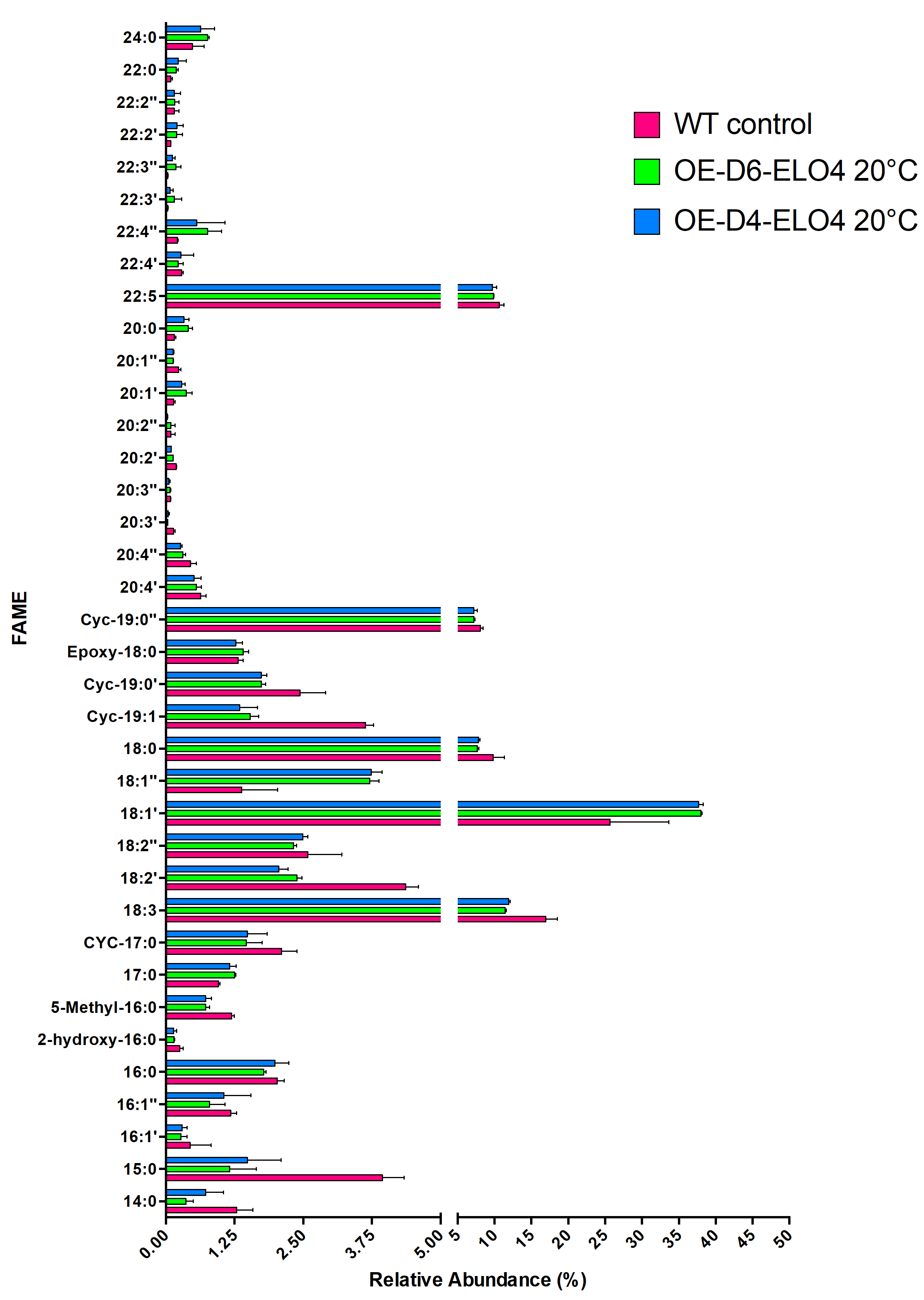


Figure S7. Fatty acid profile of genetically manipulated *C. fasciculata* cells overexpressing Cf-Δ6 or Cf-Δ4 in conjunction with Cf-Elo4 grown in standard media at 20˚C. The bar chart shows the FAs (Y axis, the order follows increasing retention time) and the relative abundance (X axis) found in *C. fasciculata* overexpressing Cf-Δ6 or Cf-Δ4 in conjunction with Cf-Elo4 (OE-D6-Elo4 and OE-D4-Elo4) and WT control, grown in standard media at 20˚C. Values are the mean of three independent biological replicates (n=3). Error bars represent the standard deviation of each mean (±). All FAs were identified using GC-MS based upon retention time, fragmentation, and comparison with standards. Statistical analysis was performed by PRISM 6 using One-way ANOVA multiple comparisons based on a Tukey t-test with a 95% confidence interval.
